# Physics-guided probabilistic modeling of extreme precipitation under climate change

**DOI:** 10.1038/s41598-020-67088-1

**Published:** 2020-06-24

**Authors:** Evan Kodra, Udit Bhatia, Snigdhansu Chatterjee, Stone Chen, Auroop Ratan Ganguly

**Affiliations:** 1 risQ, Inc., 55 Magazine St 6B, Cambridge, 02139 USA; 2 Civil Engineering, Indian Institute of Technology, Gandhinagar, 382355 India; 3 School of Statistics, University of Minnesota, Minnesota, MN 55455 USA; 4 Civil and Environmental Engineering, Northeastern University, Boston, 02115 USA

**Keywords:** Climate change, Mathematics and computing, Statistics

## Abstract

Earth System Models (ESMs) are the state of the art for projecting the effects of climate change. However, longstanding uncertainties in their ability to simulate regional and local precipitation extremes and related processes inhibit decision making. Existing state-of-the art approaches for uncertainty quantification use Bayesian methods to weight ESMs based on a balance of historical skills and future consensus. Here we propose an empirical Bayesian model that extends an existing skill and consensus based weighting framework and examine the hypothesis that nontrivial, physics-guided measures of ESM skill can help produce reliable probabilistic characterization of climate extremes. Specifically, the model leverages knowledge of physical relationships between temperature, atmospheric moisture capacity, and extreme precipitation intensity to iteratively weight and combine ESMs and estimate probability distributions of return levels. Out-of-sample validation suggests that the proposed Bayesian method, which incorporates physics-guidance, has the potential to derive reliable precipitation projections, although caveats remain and the gain is not uniform across all cases.

## Introduction

Probabilistic projections of precipitation under climate variability and change are necessary to inform water resources planning and management, design and operations of hydraulic infrastructures, and the nexus of water with food and energy^1–3^. Uncertainty assessments associated with predictive insights on precipitation extremes are particularly important for flood resilience and risk assessments^4,5^. The primary sources of uncertainties in future climate projections at stakeholder-relevant scales include our inability to project greenhouse gas emissions conditioned on social and technological change, gaps in our understanding of climate science as reflected in computer models and their parameters, natural or intrinsic variability of the climate system, and challenges in translating or downscaling larger-scale climate model simulations to the higher resolutions useful for stakeholders^6,7^. Emission trajectories are interpreted as what-if decision scenarios and as projections rather than predictions, and ensembles of model runs based on multiple such trajectories attempt to capture the range of variability in this context. While it is difficult to cast this variability in traditional probabilistic settings, prior literature has examined this variability in great detail. Intrinsic or natural climate variability is assumed to be captured through initial condition ensembles (for given model and forcing), and may be best characterized through nonlinear dynamical measures. While a probabilistic description may be possible, uncertainty characterization for systems that are sensitive to initial conditions is an ongoing research area^8^. Uncertainties in the downscaling process are challenging to characterize as well. Owing to computational resource requirements, dynamical downscaling approaches typically cannot even consider the range of plausible projections encapsulated in earth system model ensembles^9^. Meanwhile, statistical downscaling cannot readily consider uncertainties derived from assuming data-driven function mappings that may need to change as regional or global climates change in the future. While uncertainty quantification in downscaling may still be attempted^10,11^, as a matter of practice, uncertainty assessments are not yet disseminated with downscaled data products. A primary technical inhibitor is often the difficulty in comparing a climate resilience or adaptation investment’s cost to its estimated benefit (often reduced risk in economic terms). Without robust uncertainty bounds around physical climate risk projections, stakeholders are ill-equipped to confidently assess the net present value of an investment in terms of avoided worst case consequences.

While all of these areas relate to comprehensive uncertainty characterization in the context of translating earth system model simulations to credible stakeholder-relevant information, and require significant advances in the state-of-the-art and best practice, the focus of this manuscript is on characterizing gaps in the physics through model-based simulations.

Based on an implicit assumption that the range of parametric and structural variations embedded in climate models may capture the range of physically plausible behavior^12,13^, multiple model ensembles are used to characterize the variability among models and for associated uncertainty assessment. In a desire to balance two competing views, one that argues that historical skills of model simulations based on comparisons with observations is a valid indicator of future behavior, and another that suggests that given expected changes in the earth’s radiative balance model consensus is a better indicator^14^, recent research has examined the principled ways to consider both skills and consensus in a Bayesian framework^15–19^. The hypothesis that known physics-based relations that may or may not be well captured in earth system models, as well as observed and simulated data-driven multivariate dependence structures may improve this aspect of model assessment and probabilistic modeling has been suggested^20^. Here we examine this hypothesis in the context of climate model-driven probabilistic modeling of future precipitation extremes, based on temperature dependence. While we do not consider the full range of temperature scaling based on Clausius-Clapeyron and convective processes^21–23^, we do consider a specific case study. Our findings may lead to more effective uncertainty characterization, as well as better understanding of model strengths and the applicability of scientific understanding beyond what may be captured in models to improve uncertainties.

## Skill, consensus, and physics-guided climate model weighting

The most common and practical approach for probabilistic climate modeling involves exploiting archived ensembles of ESM runs to estimate probability distributions of climate change. Several methodologies for creating such ensembles have been proposed that essentially focus on skill- and consensus-based weighting of the ensemble members. Skill refers to the ability of an ESM to replicate historical climate observations, while consensus relates on their agreement with their peers about the future^14^. This approach was formalized for regional average temperature and precipitation in a Bayesian framework^15,16^. It was then extended in several studies to accommodate bivariate relationships between averages of climate variables^17^ and to support efficient probabilistic modeling across multiple geographic regions simultaneously^18^. To date, most of these studies have only supported averages of climate variables. An exception is a recent study that applies this framework to high quantiles of precipitation^19^. Specifically, it applies a modified version of the framework to the 95^*th*^ percentile of precipitation depth on wet days.

Literature has pointed out the difficulty of measuring the “skill” of an ESM^12,13,24,25^, despite a multitude of attempts to do so^26–28^. Furthermore, in many cases common skill metrics such as root mean squared error^26^ tend to not lead to systematic differences in terms of model projections^27,28^; that is, a “better model” often does not say anything different about the future than a “bad” model. Several notable studies, however, suggest that skill metrics designed to capture whether an ESM is simulating a non-trivial physical process can lead to clearer insights about anthropogenic attribution^29^ or reduced future uncertainty^23,30,31^. From this, we can synthesize a hypothesis that non-trivial, physics-guided measures of skill may be more useful indicators of ESM reliability. This hypothesis is tested formally via the Bayesian model proposed in the current study, using precipitation extremes as a case.

## Physics of precipitation extremes

Precipitation extremes are in many cases expected to increase in intensity, duration, and/or frequency as a function of climate change given theory^21,22,32^, evidence from observations^33^, and ESM projections^34,35^. At a global scale this can be explained by the Clausius Clapeyron (CC) equation^34,36^, which shows that under ideal conditions, atmospheric moisture capacity increases in a warming climate.

The August-Roche-Magnus formula^37^ provides an empirically derived approximation in ideal conditions (between −40 and 50 degrees Celsius and over a plane surface of water):

1 $${e}_{s}(T)=6.1094\exp \left(\frac{17.626T}{T+243.04}\right)$$

where *e*_*s*_ is saturation vapor pressure (i.e., atmospheric moisture holding capacity) in hPa and *T* is temperature in Celsius. Moisture condenses to precipitable water when atmospheric moisture holding capacity is reached.

On average, this implies a shift in the *distribution* of the intensity of precipitation events: larger *e*_*s*_(*T*) values imply longer duration between condensation and thus precipitation events. When heavy precipitation events do occur, they are expected to increase in intensity owing to increased atmospheric moisture content. Moreover^38,39^, revealed that super CC scaling (typically of order of 1.5–2 times than CC scaling) is primarily due to response of convection to increase in near-surface humidity, while other atmospheric conditions remain constant. Ultimately, in aggregate, increasing temperatures under climate change translates to increased capacity for drought risk with simultaneous increased potential for extreme precipitation and flood risk^40^. At a global average scale, it has been estimated that atmospheric moisture capacity increases by 7% per degree Celsius^36^; this is often referred to as CC scaling.

Generally it would be difficult to assess an ESM’s ability to simulate the dynamical processes (upward vertical wind velocities) that partially drive extreme precipitation since observational data for those processes are usually not even available. In contrast, in many regions of the world, high quality observations for both temperature and precipitation do exist.

Hence, in this study, we leverage this knowledge with the following hypothesis: a skillful ESM should be able to successfully replicate not only the observed marginal distribution of extreme precipitation but also its observed dependence (whether the relationship is positive, inverse, or lack thereof) on contemporaneous air temperature at a regional scale. The complexity of the relationship between air temperature and extreme precipitation^41^ as well as the relative regional dominance of dynamical processes^21,22,42–44^ inhibits straightforward CC based extrapolation. This further supports the potential utility in modeling the relationship between temperature and extreme precipitation at a regional scale. In other words, rather than imposing CC scaling or any specific magnitude of scaling, we allow the Bayesian model to learn the specific regional dependence between daily extreme precipitation and same-day temperature. For this study, we restrict the type of temperature-precipitation dependence to a linear type with unknown direction and magnitude. We do so while acknowledging that in many cases that dependence structure could be of other forms (monotonic but nonlinear, non-monotonic, etc.) based on the above physics discussion. Future studies could seek to extend the way the following proposed model measures that dependence.

## Methods

### The data and preprocessing steps

An ensemble of 15 ESMs from the Coupled Model Intercomparison Project Phase 5 (CMIP5) archive is used in this study. For the years 1950–1999, historical ESM runs are used (SI Table 1 and SI Table 2). For the years 2065–2089, runs from the greenhouse gas scenario RCP8.5 are used. The model presented shortly is run for all 18 continental U.S. Hydrologic Unit 2 (HU2) watersheds provided by the United States Geological Survey’s Watershed Boundary Dataset (USGS WBD)^45^. We include the metadata on the ESMs and watersheds used in the supplementary material.

USGS WBD HU2 shape files are used to identify grid cells that belong to each watershed (SI Table 3). In each watershed and for each ESM, preprocessing is conducted as follows. For each month and year, we find the day with the maximum total precipitation depth over the entire watershed. Then, for that same day, precipitation and temperature are extracted at each grid cell for that day and then averaged over grid cells in the watershed. We refer to those watershed-averaged precipitation values as block maxima, i.e., maximum values of precipitation over discrete temporal blocks, in accordance with extreme value analysis literature^46^. For each month separately, those block maxima are then sorted in ascending order and treated as return levels. Sorting block maxima in ascending order helps alleviate the fact that ESMs are likely to be out of phase with each other and observations. This idea has been utilized in statistical downscaling; with asynchronous regression approaches, the order statistics of observations are regressed on the order statistics of an ESM to create transfer functions that can be carried forward to future ESM simulations^47^. Surface (2-meter) air temperature averaged over the same days as the block maxima are extracted and re-sorted according to precipitation ordering, as well. Observational precipitation maxima and surface air temperature are extracted from a higher resolution $(\frac{1}{16})$ degree gridded observational data product^48^ for the years 1950–1999 and are preprocessed in the same manner as the ESMs.

We denote *P* as return levels/depths of precipitation and *T* as temperature averaged over the same day in the same location. The subscript *k* indexes observational datasets (there is only one observational dataset used in this study, but the Bayesian model allows for more than one); *m* ∈ [1, …, *M* = 12] indexes season (calendar month in this study), *q* ∈ [1, …, *Q* = 25] indexes the ranks of the return levels (i.e., indexes return periods) from smallest to largest from a historical climatology; *q*′ ∈ [1, …, *Q*′ = 25] the same but for the future climatology; *j* indexes ESM datasets.

Let *Z*_*j,m*_ = log(*P*_*j,m,q*=1_), i.e., for any ESM dataset *j* (or *k* for observations), the smallest value of the precipitation is transformed with a natural log. Then, for larger return levels *q* ∈ [2, …, *Q*], we let *U*_*j,m,q*_ = log(*P*_*j,m,q*_ − *P*_*j,m,q*−1_), *i.e*., the logged difference between adjacent return levels. We note that while using logged difference, which essentially models a quantile process as a random walk with log-normal increments, is slightly unusual; we decided on using this framework primarily for computational convenience. This preprocessing is done for three separate climatologies: 1950–1974, 1975–1999, and 2065–2089. For the *historical period* we use the data from 1950–1974 for model estimation and the data from 1975–1999 to validate our prediction. Then using the information based on such model fitting over a historical data period and an optimized model fitted to the data from 1975–1999, we project probabilistic precipitation extremes scenarios in 2065–2089.

### The bayesian ensemble model

We leverage the Bayesian skill and consensus-based framework discussed earlier^15–19^ as the mechanical foundation for our model. Through a Markov Chain Monte Carlo (MCMC) process, ESM projections of return levels are iteratively weighted and averaged according to (1) their skill as measured by their similarity to observational return levels and (2) their consensus with projections. Skill is formulated to explicitly evaluate whether the return levels from ESMs depend on temperature in the same way that they do in observations. SI Tables 4 and 5 summarizes all the notations used in this research.

First, the smallest of the return levels are assumed to follow Gaussian data models:

2 $${Z}_{k,m} \sim N({C}_{m},{({\tau }_{k}{\sigma }_{k})}^{-1}),$$

3 $${Z}_{j,m} \sim N({C}_{m}+CBIA{S}_{j},{\sigma }_{j}^{-1}),$$

4 $${Z{\prime} }_{j,m} \sim N({C{\prime} }_{m}+CBIA{S}_{j},{(\theta {\sigma }_{j})}^{-1}).$$

Here, the unknowns *C*_*m*_ and ${C{\prime} }_{m}$ are seasonal parameters that can be estimated given that there are multiple models and observational datasets. *CBIAS*_*j*_ is a bias term for ESM *j* that is assumed to be constant over time regimes. The parameter *σ*_*j*_ is a scalar weight for each ESM. Finally, *θ* is a future variance scaling parameter that modulates the importance of consensus in the determination of weights and also allows for a different magnitude of uncertainty in the future climatological regime^20^. Similar to models from past studies^15,16,18^, the weight parameter *σ*_*k*_ is estimated from observational data as the inverse of the sample variance of the smallest block maxima (*q* = 1) over all *M* seasons.

Define the variable *δ*_*j,m,q*_ = *T*_*j,m,q*_ − *T*_*j,m,q*−1_ for any ESM *j* (or observational dataset *k*). Similar to past related studies^15,16,18^, we fix the parameter ${\varepsilon }_{k,q}^{-1}$ as follows: first, separately for each season *m*, we fit a simple linear regression of ${U}_{k,m,q\in [2,\ldots ,Q]}$ on ${\delta }_{k,m,q\in [2,\ldots ,Q]}$. We save the residuals from each of these regressions. Then, for each order statistic *q*, we calculate the sample variance of the residuals for using *q* all seasons *m* ∈ [1, …, *M*] Using these, we define the data model for values of *U*_*w,m,q*_ and ${U{\prime} }_{w,m,q{\prime} }$, for *q* ∈ [2, …, *Q*] and *q*′ ∈ [2, …, *Q*′].

5 $${U}_{k,m,q} \sim N({\gamma }_{m,q}+{\phi }_{m}{\delta }_{k,m,q},{({\tau }_{k}{\varepsilon }_{k,q})}^{-1}),$$

6 $${U}_{j,m,q} \sim N({\gamma }_{m,q}+{\alpha }_{j,m}+{\phi }_{m}{\delta }_{j,m,q},{({\varepsilon }_{j,q})}^{-1}),$$

7 $${U{\prime} }_{j,m,q{\prime} } \sim N({\gamma {\prime} }_{m,q{\prime} }+{\alpha }_{j,m}+{\phi {\prime} }_{m}{\delta {\prime} }_{j,m,q{\prime} },{({\beta {\prime} }_{m,q{\prime} }{\varepsilon }_{j,q})}^{-1}).$$

The treatment of *σ*_*k*_ and *ε*_*k,q*_ as fixed and estimated from data is the empirical aspect of the Bayesian model proposed here. Empirical Bayesian methods leverage the Bayesian statistical paradigm to obtain posterior distributions of unknown parameters conditional on data, but with considerably less intricate computations and generally near identical theoretical properties.

With Eqs. 5–7, we are essentially assuming that the logarithm of the differential between any pair of subsequent order statistics, which is a sample quantity, is Gaussian with the mean being the population equivalent. As such, the model does not suggest that extremes themselves are Gaussian, it merely says sample versions are normally distributed around true population quantities. This Gaussian assumption of the *U*_*k,m,q*_ statistics is examined in greater detail in the supplementary materials. It is important to keep in mind that observations themselves are potentially noisy realizations of the truth. Our Bayesian model does not necessarily treat observations as ground truth in the way a supervised learning approach would. The parameter *τ*_*k*_ lets us scale the weight of observational climate data to behave more like ground truth, which in turn influences values of unknown parameters in a manner similar in spirit to a supervised learning problem. We explore the sensitivity of model results to choice of *τ*_*k*_ in the supplementary material, based on which we settle on *τ*_*k*_ = 100 for our data analysis in this paper.

The full joint distribution of all the unknown parameters: *C*_*m*_, ${C{\prime} }_{m}$, *CBIAS*_*j*_, *σ*_*j*_, *θ*, *ϕ*_*m*_, *α*_*j.m*_, *ε*_*j,q*_, *γ*_*m,q*_, ${\gamma {\prime} }_{m,q{\prime} }$, ${\phi {\prime} }_{m}$, and ${\beta }_{m,q{\prime} }$ is not of an analytically known form. Similar to past studies^15,16,18^, we choose conjugate prior distributions for each unknown that lead to known full conditional posterior distributions. All unknowns are updated in a Gibbs sampler variant of a Markov Chain Monte Carlo (MCMC) simulation. In the supplementary materials, we provide full details on the prior parameters, sensitivity tests for key prior parameters, the full conditional posterior distribution for all unknowns, MCMC simulations and associated diagnostics. SI Tables 6–8 summarizes the selected priors and parameter starting values.

Validation of the Bayesian model is a crucial component of assessing its utility. Of course, unlike weather forecasting, true validation over future climatologies is impossible in the immediate term given the lead times of interest. We validate the model using a training-holdout scheme similar to conventional predictive modeling. We do this in each region using 1950–1974 as the “training” and 1975–1999 as the “validation” climatologies, respectively. We examine the accuracy of our Bayesian model, posterior coverage, posterior upper coverage, and posterior width, all as compared to the original ensemble of ESMs. In addition, we also compare posterior projected *changes* in return levels as compared to those projected changes obtained directly from the original ensemble of ESMs. For this measure, where the original ensemble performs well with reference to held out observations, the ideal Bayesian model should exhibit similar projected changes. In cases where the original ensemble performs poorly against held out observations, the ideal Bayesian model might deviate in terms of projected changes.

Finally, similar in theme to related work^18^, we use the ESMs themselves to validate the model. For a given watershed, each ESM is iteratively treated as true climate and the Bayesian model is run using 1975–1999 as the training climatology and 2065–2089 as the validation climatology. This is motivated by the fact that the difference between 2065–2089 and 1975–1999 should show a more prominent signal and thus might be a more fair way to assess the ability of the Bayesian model to handle longer-term changes (than those between 1950–1974 and 1975–1999) and potential nonstationarity. Details of these validation steps are reported in the supplementary materials.

## Results

### Validation results

**Figure 1:**
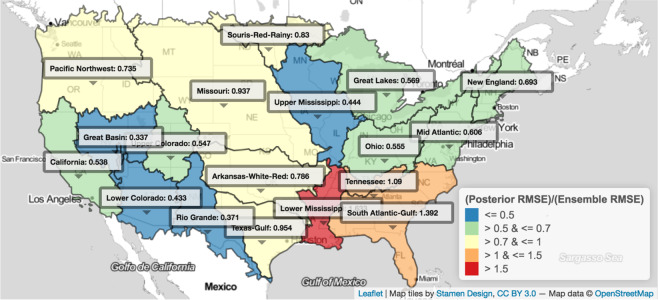
The performance of the Bayesian model is compared to using the raw ensemble in terms of out-of-sample accuracy and predictive coverage across 18 watersheds that comprise the continental U.S. Coloring represents accuracy of the posterior relative to using an ensemble average approach, measured as $\frac{RMS{E}_{p}}{RMS{E}_{e}}$. Accuracy is higher in 15 out of 18 watersheds. In 15 of 18 watersheds, using a 99% credible interval, posterior coverage is larger than or equal to than ensemble coverage in all watersheds, where coverage ranges from 0 to 1 (not depicted). The three regions where posterior coverage is smaller than that of the original ensemble are the Tennessee, Pacific Northwest, and California watersheds. Upper coverage is equivalent or improved in only 3 out of 18 watersheds using the same 99% credible interval, including Lower Mississippi, Texas-Gulf, and Upper Mississippi. Watersheds are labeled by name and their respective $\frac{RMS{E}_{p}}{RMS{E}_{e}}$ values. (We use R package that wraps around the open source Javascript project Leaflet: https://rstudio.github.io/leaflet/ to create the figure).

Figure 1 displays validation scheme accuracy results across the 18 USGS HU2 watersheds that comprise the continental United States on a map (See SI Figs. 1–3 for validation, cross-validation and average distance from ensemble mean). Out-of-sample RMSE-based accuracy, posterior coverage, posterior upper coverage, and posterior distribution width are tabulated for each watershed in Table 1 (See SI Figs. 4–6 for prior and posterior distributions for validation scheme model runs for sample (Ohio) watershed). In the majority of watersheds (15 of 18), the Bayesian model outperforms the equal weighted ensemble average relative to held out observations from 1975–1999 in terms of RMSE-based accuracy. For 16 of 18 watersheds, the Bayesian model equals or outperforms the equal weighted ensemble upper and lower bounds in terms of posterior coverage when using a 99% posterior credible interval. Marginally, the Bayesian model outperforms the ensemble in 81.8% of the return levels corresponding to *q*′ ∈ [1, …, 25] in the 18 watersheds (averaged over the months), and in 73.1% of the months (averaged over the return levels).

**Table 1:** Watershed level validation metrics are tabulated.

Watershed	*RMSE*_*p*_	$\frac{RMS{E}_{p}}{RMSEe}$	*cov*_*p*_	$\frac{co{v}_{p}}{co{v}_{e}}$	$co{v}_{p}^{u}$	$\frac{co{v}_{p}^{u}}{co{v}_{e}^{u}}$	*W*_*p*_	$\frac{{W}_{p}}{{W}_{e}}$
Arkansas-White-Red	2.16	0.79	0.98	1.08	0.98	0.98	11.37	1.12
California	2.19	0.54	0.92	1.01	0.97	0.97	9.38	0.73
Great Basin	1.67	0.34	0.79	1.55	0.93	0.93	4.71	0.49
Great Lakes	2.12	0.57	0.88	1.69	0.96	0.96	5.71	0.78
Lower Colorado	2.13	0.43	0.83	0.95	0.92	0.92	5.24	0.38
Lower Mississippi	5.75	1.63	0.99	1.00	0.99	1.00	36.16	2.20
Mid Atlantic	4.33	0.61	0.95	1.34	0.96	0.96	10.68	0.69
Missouri	1.89	0.94	0.87	1.25	0.97	0.97	6.17	1.09
New England	5.02	0.69	0.89	1.73	0.89	0.89	10.62	0.65
Ohio	2.81	0.56	0.98	1.58	0.99	0.99	12.15	0.97
Pacific Northwest	1.54	0.73	0.98	1.16	0.98	0.98	7.47	1.18
Rio Grande	1.71	0.37	0.90	1.72	0.98	0.98	5.07	0.64
Souris-Red-Rainy	2.65	0.83	0.68	1.31	0.85	0.85	4.71	0.53
South Atlantic-Gulf	3.77	1.39	0.95	0.97	0.95	0.97	13.06	1.06
Tennessee	5.92	1.09	0.96	1.02	0.96	0.96	20.50	0.76
Texas-Gulf	3.19	0.95	0.95	1.07	0.95	1.01	10.01	0.87
Upper Colorado	1.72	0.55	0.88	1.39	0.96	0.96	4.71	0.63
Upper Mississippi	1.60	0.44	0.99	1.25	1.00	1.00	8.54	0.81

Bayesian accuracy is shown on its own (*RMSE*_*p*_) and relative to the original ensemble as a ratio $(\frac{RMS{E}_{p}}{RMSEe})$. The same is tabulated for coverage $(co{v}_{p}\,\mathrm{and}\,\frac{co{v}_{p}}{co{v}_{e}})$, upper coverage $(co{v}_{p}^{u}\,\mathrm{and}\,\frac{co{v}_{p}^{u}}{co{v}_{e}^{u}})$, and width $({W}_{p}\,\mathrm{and}\,\frac{{W}_{p}}{{W}_{e}})$.

Overall, the Bayesian model tends to be more accurate than the ensemble in non-summer months. Note that ESMs poorly simulate the pronounced diurnal cycle in precipitation over the United States in the summer^49^, and there is a significant correlation between tropical and North Pacific sea surface temperatures and summer precipitation variability^41^. Using these factors in the Bayesian model can potentially yield even better comparative performance over the unweighted ensemble. However, we did not pursue such more mathematically complex models to keep the analysis tractable and interpretable.

**Figure 2:**
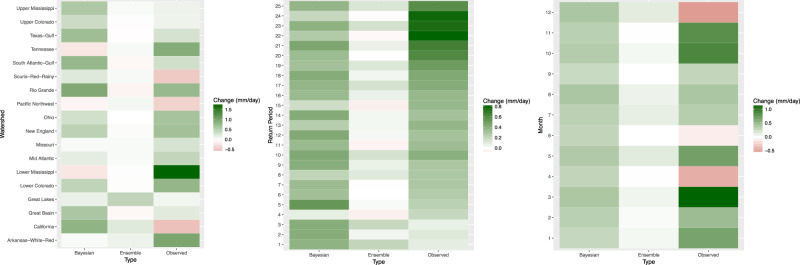
(Left) In each watershed, median historical Bayesian changes are calculated (median of the 1975–1999 minus the 1950–1974 climatology), where the median of the 1950–1974 posterior is subtracted from the median of 1975–1999. Those changes are averaged across all return periods and seasons. The same changes are calculated for the median of changes from the original ensemble and from observed changes. Brown serves to indicate decrease, green increase, and white means no change. (Center) The same as the left panel but averaged across watersheds and seasons. (Right) The same as the left panel but averaged across watersheds and return periods.

Figure 2 examines the ability of the Bayesian model to simulate historical changes in return levels compared to the original ensemble projections. Historical changes (1950–1974 to 1975–1999) are shown for the Bayesian model, the original ensemble, and the observations. All changes are in $\frac{mm}{day}$ units and are separately examined marginally over watersheds, return periods, and seasons. Median projections from the Bayesian model better reflect observed magnitudes of change, whereas the median of the original ensemble consistently underestimates observed change. The relative consistency of the Bayesian projections, versus those obtained from the raw ensemble, may reflect a combination of (1) the Bayesian model’s ability to weight ESM projections more realistically using observations and/or (2) “shrinkage”, or the process of the model parameters tending toward average behavior over seasons and order statistics.

### Projections

**Figure 3:**
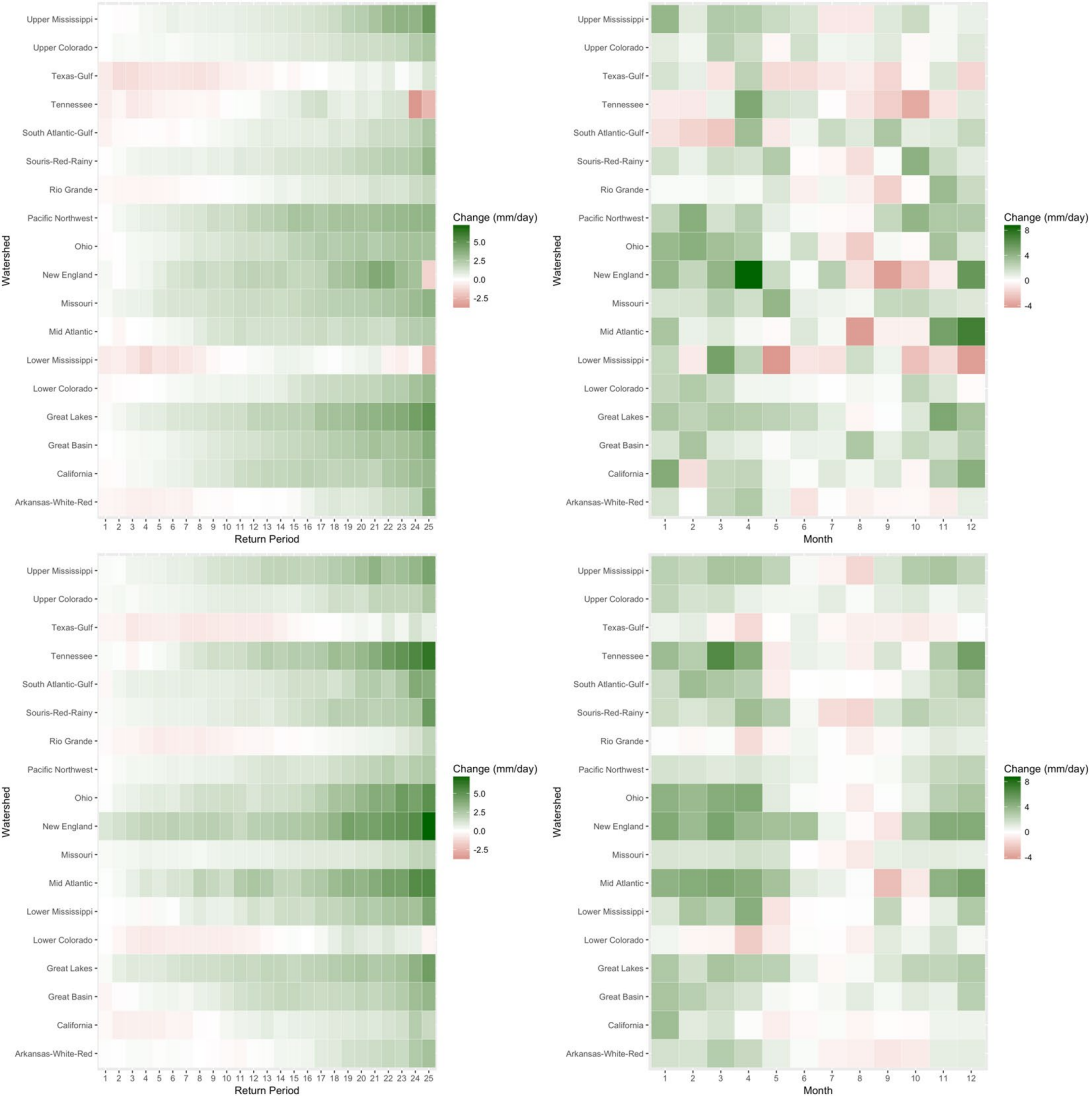
(Top left) Similar to Fig. 2, median projected changes (1975–1999 to 2065–2089) are shown for the Bayesian model for each return period and watershed, averaged over all months. (Bottom left) The same is shown as the top left but for the medians of the original ensemble. (Top right) Median projected changes are shown for the Bayesian model for each season, averaged over all return levels. (Bottom right) The same is shown as the bottom left but for the medians of the original ensemble.

Figure 3 provides a comprehensive look at median change projections from both the Bayesian model and the original ensemble, but without information on uncertainty. Heatmaps show medians of posterior projections and the original ensemble for all combinations of return levels and watershed, averaged over all calendar months in each case. Results for both the original ensemble and the Bayesian model in Fig. 3 resemble what could be generally expected under CC scaling in every watershed, where progressively further into the upper tail of the extreme precipitation distribution, intensity increases more^34^. For the Bayesian model, ~82% (369 of 450) of heatmap cells show increases in return levels, compared to ~85% (383 of 450) for the original ensemble.

Figure 3 also shows the same but show median projected changes for each month, averaged over all return levels, for the Bayesian model and original ensemble, respectively. Here, ~72% (156 of 216) of cells show an increase for the Bayesian model, whereas ~76% (165 of 216) do for the original ensemble. The seasonal pattern of change is similar for both Bayesian and original ensemble projections, with June through September showing more cases of average decrease and the rest of the year showing increases more frequently across the majority of watersheds.

**Figure 4:**
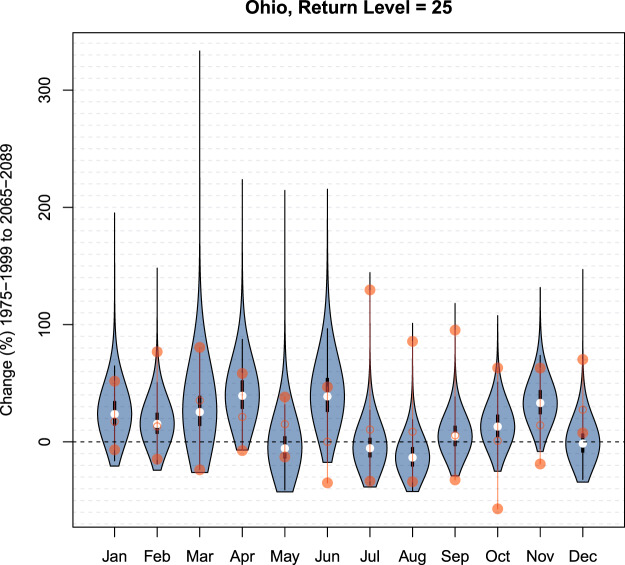
Blue violin plots depict kernel densities of Bayesian probability distributions of projected change (1975–1999 to 2065–2089) in *q*′ = 25-year return levels in the Ohio watershed for each month. White dots represent the median of the Bayesian posteriors, and thick and thin black whiskers are lower and upper fences seen in a standard boxplot. Red hollow dots represent the median of the original ensemble projected changes. Red filled dots represent the upper and lower bounds of the original ensemble. Fences of the violin plot represent the kernel density functions of Bayesian probability distributions for each month.

We now focus on one watershed, Ohio, as a complete case study. Figure 1 shows that the Bayesian model substantially improves on its accuracy in the 1975–1999 time period, cutting RMSE almost in half. Figure 2 shows that the Bayesian model also more realistically portrays the change in average Ohio extremes. Figure 3 shows that, qualitatively, the Bayesian model yields similar projected changes as the median projected changes from the original ensemble. Table 1 shows that, while the Bayesian model cuts RMSE almost in half, it also effectively on average reduces width as measured by the credible interval. Finally, we explore detailed projected changes for the uppermost extremes: Fig. 4 shows the detailed end of century percent change projections (1975–1999 median to 2065–2089) for *q*′ = 25 year return levels Ohio. Violin plots show a full probability distribution of change relative to the 1975–1999 from the Bayesian model for each calendar month. Median, lower bound, and upper bound change projections from the original ensemble are overlaid for comparison. One notable feature in the Bayesian projections that is absent in the original ensemble is a long upper tail. Another is the difference between median projections of the Bayesian model versus the original ensemble, which can provide insight into biases being estimated. December projections show this difference, where the full spread of ESM projections shows increased precipitation, but the Bayesian model shows a median of projected decrease but a heavy upper tail. This lends an explicit likelihood to potential changes that are larger than the original ensemble projections. Though generally similar on average, the Bayesian change projections have the advantage over the original ensemble in that they provide stakeholders with information on probabilities versus discrete, unweighted projections.

### Significance of temperature as a covariate

One of the principal hypotheses of this study is that guiding the statistical architecture of the model with known physics could enhance the results, potentially in a number of ways. In this case the hypothesis centers on the inclusion of same day temperature as a covariate. To test this, we run an experiment with one variant evaluating the model’s performance in terms of RMSE performance, posterior coverage, posterior upper coverage, posterior distribution width (all of of ${\gamma {\prime} }_{m,q{\prime} }$) while including *ϕ*_*m*_ and ${\phi {\prime} }_{m}$ as random unknowns and another variant where we set ${\phi }_{m}={\phi {\prime} }_{m}=0$, effectively removing the notion of temperature dependence. We then perform a meta-analysis of the model’s performance against the validation regime with versus without temperature dependence.

Table 2 synthesizes the relative difference in model performance when including temperature dependence versus not including it. Including temperature dependence improves overall RMSE in 7 of 18 watersheds, increases or maintains coverage in 11 of 18, increases or maintains upper coverage in 14 of 18, and increases average posterior width in 11 of 18. In the Lower Mississippi, South Atlantic-Gulf, and Ohio watersheds, accuracy improves notably with temperature dependence included. There appears to be a mild bias-variance tradeoff, where posterior intervals improve (and often widen) and accuracy decreases slightly in general. The ability of the Bayesian model to capture bias of ESMs likely contributes to its performance more so than temperature dependence. Despite these mild tradeoffs when including temperature, the Bayesian model still generally outperforms the ensemble in terms of accuracy (see Fig. 1 and Table 1).

**Table 2:** Validation metric ratios are shown for RMSE, coverage, upper coverage, and width for the posterior with temperature dependence compared to without temperature dependence (denoted as *p*, *ϕ* and *p*, !*ϕ*, respectively, in the table header).

Watershed	$\frac{RMS{E}_{p,\varphi }}{RMS{E}_{p,!\varphi }}$	$\frac{co{v}_{p,\varphi }}{co{v}_{p,!\varphi }}$	$\frac{co{v}_{p,\varphi }^{u}}{co{v}_{p,!\varphi }^{u}}$	$\frac{{W}_{p,\varphi }}{{W}_{p,!\varphi }}$
Arkansas-White-Red	1.06	0.99	0.99	1.00
California	0.96	1.00	1.00	1.00
Great Basin	1.04	1.00	1.02	1.04
Great Lakes	0.93	1.04	1.01	1.02
Lower Colorado	1.02	1.01	1.01	1.00
Lower Mississippi	0.97	1.00	1.00	0.99
Mid Atlantic	1.04	0.99	0.99	1.00
Missouri	0.96	1.02	1.02	1.00
New England	1.00	0.99	0.99	0.97
Ohio	1.03	0.99	1.00	0.99
Pacific Northwest	0.99	1.00	0.99	0.98
Rio Grande	1.05	0.98	1.00	0.95
Souris-Red-Rainy	1.02	0.99	1.00	1.00
South Atlantic-Gulf	1.00	1.00	1.00	1.03
Tennessee	1.02	1.00	1.00	1.00
Texas-Gulf	1.05	1.00	1.00	1.00
Upper Colorado	0.95	0.99	1.00	0.96
Upper Mississippi	0.85	1.02	1.00	1.00

Including temperature dependence improves overall RMSE in 7 of 18 watersheds, increases or maintains coverage in 11 of 18, increases or maintains upper coverage in 14 of 18, and increases average posterior width in 11 of 18.

## Discussion

In this study, we present a physics-guided Bayesian model that utilizes ensembles of ESMs to estimate probabilistic projections of precipitation extremes under climate change. We exploit the knowledge that in many regions there is a relationship between temperature and extreme precipitation (e.g.^34^), but that the dependence structure between the two variables might often be more complex than idealistic Clausius Clapeyron scaling^50^. The Bayesian model weights ESMs according to their ability to capture not only historically observed marginal, univariate statistics of daily total precipitation return levels but also their covariance with historically observed same-day average surface temperature. This study has a similar goal to a Bayesian model for precipitation extremes developed recently^19^ but is more generalized in the sense that it simultaneously models a complete cumulative distribution function of extreme events rather than one specific statistic of extreme precipitation.

For the model specific to precipitation extremes developed here, there are several caveats worth highlighting. ESMs would not explicitly capture (extra)tropical cyclones or other major storms (e.g., Nor’easters) from the observational record, and thus extreme precipitation as a result of those types of events might not be reflected directly in raw ensemble data. This may impact the Bayesian model’s ability to accurately capture some of the most extreme observed events, especially in the southeastern United States (e.g., see Fig. 1). In the validation analysis, while the Bayesian model’s 99% credible intervals performed well overall, they did not tend to outperform the original ensemble in terms of upper coverage, i.e., the ability to bound the most extreme observations. This could be explained partially be a form of bias-variance trade-off, given that the Bayesian model usually outperforms the ensemble in terms of accuracy in the 1975–1999 period. It is also reasonable to hypothesize that a Generalized Extreme Value (GEV) data model, for example, might better handle the uppermost extremes. Future research could expand the development of tractable, intepretable Bayesian frameworks for ensemble weighting based on a GEV data model^19^. For this particular work, results in Fig. 4 do show that the upper tail of projected changes can be quite heavy; this is not reflected in the 99% credible intervals.

Caution must also be exercised in interpreting Bayesian projections, especially given mixed results from the ESM cross validation experiment (see supplementary materials). Stationarity and reliability of a relatively complex multivariate distribution of Bayesian parameters, while tested to the extent possible via cross validation with ESMs and with an explicit training-holdout split in the historical time window, is not guaranteed in the future^25^. However, the Bayesian model does produce projections that are qualitatively comparable to those of the original ensemble but with probabilistic information, implying that the risk of leaning on the Bayesian model versus the raw ESMs is minimal in a relative sense.

From stakeholder perspective, near term and long term risk assessment needs to account for uncertainty from disparate sources and climate uncertainties may be of second order. For example, in case of hydrological and flood risk assessment, parametric and modelling uncertainties owing to limited understanding of underlying processes can dominate or in some cases, comparable to climate uncertainties^51^. Similarly, in the context of risk assessment on critical infrastructures including transportation, energy, and water and wastewater networks, uncertainties associated with associated non-linear dynamics and cascading failures^52^ can dominate the stressor related uncertainties. Hence, future research and methodologies in this direction need to characterize, both qualitatively or quantitatively, the relative importance and magnitude of various sources of uncertainties within mathematical frameworks to aid policymakers in decision making.

As discussed more in the supplementary materials, certain combinations of prior parameters may work particularly well in certain watersheds, but in this study we opted to find one set of parameters that worked well, generally (See SI Figs. 7–8). The Bayesian model generally performs best when skill is favored over consensus ((See SI Figs. 9–17 for watershed scale results). This may suggest that in general, weighting ESMs based on nontrivial and physics-guided measures of historical skill (in this case, how well ESMs portray precipitation-temperature dependence from observed data) can lead to improvements in the statistical attributes of probabilistic projections, e.g., accuracy and coverage.

## Supplementary information

Supplementary Information.
