# Supplementary material for: Physics-guided probabilistic modeling of extreme precipitation under climate change
Source: Sci Rep. 2020 Jun 24;10:10299. doi: 10.1038/s41598-020-67088-1 (PMC7314860; doi:10.1038/s41598-020-67088-1)
Supplement: Supplementary file 1 — Supplementary Information. [file 41598_2020_67088_MOESM1_ESM.pdf]

Supporting Information for
"Physics-guided probabilistic modeling of extreme precipitation under climate change"

Evan Kodra, Udit Bhatia, Snigdhansu Chatterjee, Stone Chen, Auroop Ratan Ganguly

1 Data and Preprocessing: Additional Detail

Table 2 summarizes the ensemble of 15 ESMs from the CMIP5 archive used in this study. For the years 1950-1999, historical ESM runs are used. For the years 2065-2089, runs from the greenhouse gas emissions scenario Representative Concentration Pathway (RCP) 8.5 are used. The Bayesian model presented in the main text is run for all 18 continental U.S. Hydrologic Unit 2 (HU2) watersheds provided by the United States Geological Survey's Watershed Boundary Dataset (USGS WBD) [*Berelson et al.*, 2004]. Table 3 lists and provides a short identification number for each of the 18 watersheds.

2 Prior distributions for the Bayesian analysis

Conjugate prior distributions are defined as follows:

$$P(C'_m) \sim N(C_0, \sigma_0^{-1}) \tag{1}$$

$$P(CBIAS_j) \sim N(\delta_0, \rho_0^{-1}) \tag{2}$$

$$P(\sigma_j) \sim G(\alpha_0, \beta_0) \tag{3}$$

$$P(\theta) \sim G(\zeta_0, \eta_0) \tag{4}$$

Corresponding author: Udit Bhatia, `bhatia.u@iitgn.ac.in`

$$P(\gamma'_{m,q'}) \sim N(\gamma_0, \epsilon_0^{-1}) \tag{5}$$

$$P(\alpha_{j,m}) \sim N(\nu_0, \omega_0^{-1}) \tag{6}$$

$$P(\phi_m, \phi'_m) \sim N(\phi_0, \xi_0^{-1}) \tag{7}$$

$$P(\epsilon_{j,q}) \sim G(\lambda_0, \upsilon_0) \tag{8}$$

$$P(\beta'_{m,q'}) \sim G(\kappa_0, \iota_0) \tag{9}$$

3 Prior Choices and Sensitivity

Our choices for priors values, with justifications if notable, are shown in Table 5. Table 6 defines the values we explore for the priors that exert relative influence, those marked with ** in Table 5. Note that these are all priors for variance scaling parameters. In Section 7, we provide more general details on the MCMC procedure. For the above experiment (where there are 625 combinations of the 4 priors parameters in total, for each watershed), we take iterations to $N_1 = 300$ for the burn-in and $N_{final} = 1,000$ after thinning.

Figure 7 shows the results from this sensitivity test. The Bayesian model accuracy is most sensitive to choices of κ_0 and ι_0, as can be seen most clearly from the top left panel. In all other experiments and in the final model runs, based on the results from this experiment, we set the 4 selected priors as: $\kappa_0 = 10$, $\iota_0 = 25$, $\zeta_0 = 1$, and $\eta_0 = 10$, for every watershed. These choices can be interpreted in post-hoc fashion to an extent. Setting $\kappa_0 < \iota_0$ means that our prior expectation is that $\beta_{m,q'} < 1$, or that consensus should be favored less than skill in choosing weights $\epsilon_{j,q}$. But the actual absolute values of these priors and the specific ratio of $\frac{\kappa_0}{\iota_0}$ that work well in terms of validation metrics appear to depend on the absolute value range of the precipitation data itself, and as such our final choices for these two parameters are informed by this experiment. Similarly, $\zeta_0 < \eta_0$ means that our prior belief is that $\theta < 1$, again that consensus should be favored less than skill in choosing weights σ_j.

It is worth noting that different choices of these selected priors, custom-selected per watershed, can lead to improved results in terms of validation. This was observed anecdotally when exploring results from this experiment. We purposefully refrained from choosing different priors per watershed in this study in an effort to not “overfit” and to avoid losing the value of having one set of interpretable priors. We also note the caveat that this experiment violates the principle of prior parameter selection, which is conventionally not supposed to be informed by data. An alternative approach could have been treating the priors themselves as random parameters with hyperpriors.

3.1 Skill versus Consensus

Comparatively, previous literature using the skill-consensus framework have suggested the possibility that consensus among ESMs about the future can receive too much emphasis relative to skill [*Tebaldi et al.*, 2004; *Ganguly et al.*, 2013]. In this study, we find that prior parameter choices that tend to produce posteriors that perform well in terms of out-of-sample validation also tend to produce values of θ and $\beta'_{m,q'}$ that are usually smaller than 1, effectively emphasizing skill over consensus. Figures 4 and 5 show posteriors for these parameters for the Ohio watershed (results are similar for all watersheds). Skill is generally favored over consensus $\sim$ 15-to-1 for $q' = 1$ on average according to θ and $\sim$ 2-to-1 for $q' \in [2, ...Q' = 25]$ according to $\beta'_{m,q'}$.

4 Leave one ESM our validation experiment

We also perform a leave-one-out cross validation exercise across all ESMs, thematically similar to related work [*Smith et al.*, 2009]. Specifically, we iteratively treat each ESM as if was an observational dataset and run the Bayesian model using the 1975-1999 and 2065-2089 climatologies. We note that cross validation is for the absolute values of precipitation extremes in 2065-2089. Figure 2 displays heatmaps of ratios of validation metrics (RMSE, coverage, upper coverage, and posterior width), where the numerator is the metric for the Bayesian model averaged over return periods and seasons, and the denominator is the same but for the original ensemble. On the whole, the original ensemble seems to perform at about the same level as the Bayesian model in this experiment. In terms of accuracy, several ESMs (e.g., inmcm4 and noresm1m) are consistently better forecasted when using the Bayesian model versus the original ensemble.

Figure 3, which characterizes each ESM's average distance from the ensemble mean (i.e., a measure of "consensus") by watershed, suggests that relative performance of the Bayesian model is related to whether an ESM tends to fall below or above the ensemble mean. Average distance from the ensemble mean is calculated for each ESM over each watershed for 2065-2089. Specifically, all precipitation depths are subtracted from the mean precipitation over all seasons and return periods for a given overall ESM and watershed average. Those differences are then averaged at per watershed and ESM. In the heatmap, brown cells are those where an ESM on average is "drier" and green wetter than the average of the ensemble. These differences are moderately and positively correlated to the RMSE (Spearman rank pattern correlation of 0.370), upper coverage (0.301), and width ratios (0.377) (but not the coverage ratio) displayed in Figure 2. This seems to be an apparent reflection of a bias-variance tradeoff within the Bayesian model that depends partially on the direction (higher or lower) and distance of (proxy) true future observations from the ensemble average. Also, We computed the Spearman rank correlation between the absolute value of the mean distance from the ensemble and the ratio of the Bayesian RMSE to the original ensemble RMSE. The correlation coefficient is -0.461; thus, as the absolute distance from the ensemble mean increases, as the reviewer hypothesizes, the Bayesian model tends to have a more significant impact on improving accuracy.

5 Observational Scaling Sensitivity Analysis

The parameter τ_k is used to manually scale the weighting of observations relative to ESMs in the Bayesian model. Figure 8 shows the results from an experiment wherein the Bayesian model is run over each watershed 5 times with the only difference being the value of τ_k: 1, 10, 50, 100 and 500. In this experiment, we set the burnin size at 500 and $N = 1000$, and we save every 5th sample after burnin for thinning. Validation metrics are examined for each of the 5 values of τ_k. In most watersheds, as τ_k increases, accuracy and coverage improve. However, upper coverage decreases moderately. There is no clear pattern for average posterior width. Coverage and accuracy asymptote at approximately $\tau_k = 100$, and so for every other MCMC run described in this study, we set $\tau_k = 100$ while noting that this entails a small tradeoff with upper coverage.

6 Posterior distributions

Full conditional posterior distributions are shown as follows:

$$P(C'_m|...) \sim N(\frac{c'}{d'}, \frac{1}{d'}) \tag{10}$$

where

$$c' = \sigma_0 C_0 + \theta \sum_j \sigma_j (Z'_{j,m} - CBIAS_j) \tag{11}$$

and

$$d' = \sigma_0 + \theta \sum_j \sigma_j \tag{12}$$

$$P(CBIAS_j|...) \sim N(\frac{g}{h}, \frac{1}{h}) \tag{13}$$

where

$$g = \rho_0 \delta_0 + \sigma_j \sum_m (Z_{j,m} - C_m) + \theta \sigma_j \sum_m (Z'_{j,m} - C'_m) \tag{14}$$

and

$$h = \rho_0 + M\sigma_j(1 + \theta) \tag{15}$$

$$P(\sigma_j|...) \sim G(g, h) \tag{16}$$

where

$$g = \alpha_0 + M \tag{17}$$

and

$$h = \beta_0 + 0.5 \sum_m ((U_{j,m,q=1} - C_m - CBIAS_j)^2) + \tag{18}$$

$$0.5\theta \sum_m ((U'_{j,m,q'=1} - C'_m - CBIAS_j)^2) \tag{19}$$

$$P(\theta|...) \sim G(n, o) \tag{20}$$

where

$$n = \zeta_0 + 0.5J + 0.5M \tag{21}$$

and

$$o = \eta_0 + 0.5 \sum_{j,m} (\sigma_j (U'_{j,m,q'=1} - C'_m - CBIAS_j)^2) \tag{22}$$

The next posteriors for $q > 1$ and $q' > 1$ can be estimated completely independently of those above for $q = q' = 1$, which can be advantageous computationally:

$$P(\gamma'_{m,q'}|...) \sim N(\frac{p'}{r'}, \frac{1}{r'}) \tag{23}$$

where

$$p' = \epsilon_0\gamma_0 + \beta'_{m,q'}\sum_j \epsilon_{j,q}(U'_{j,m,q'} - \alpha_{j,m} - \phi'_m\delta'_{j,m,q'}) \tag{24}$$

and

$$r = \epsilon_0 + \beta'_{m,q'}\sum_j \epsilon_{j,q} \tag{25}$$

$$P(\phi_m|...) \sim N(cc, dd) \tag{26}$$

where

$$cc \quad = \quad \xi_0\phi_0 + \sum_{k,q}\tau_k\epsilon_{k,q}(\delta_{k,m,q})(U_{k,m,q} - \gamma_{m,q}) + \tag{27}$$

$$\sum_{j,q}\epsilon_{j,q}(\delta_{j,m,q})(U_{j,m,q} - \gamma_{m,q} - \alpha_{j,m}) \tag{28}$$

and

$$dd = \xi_0 + \sum_{k,q}\tau_k\epsilon_{k,q}(\delta_{k,m,q})^2 + \sum_{j,q}\epsilon_{j,q}(\delta_{j,m,q})^2 \tag{29}$$

$$P(\phi'_m|...) \sim N(cc', dd') \tag{30}$$

where

$$cc = \xi_0\phi_0 + \sum_{j,q'}\beta'_{m,q'}\epsilon_{j,q}(\delta_{j,m,q'})(U'_{j,m,q'} - \gamma'_{m,q'} - \alpha_{j,m}) \tag{31}$$

and

$$dd = \xi_0 + \sum_{j,q'}\beta'_{m,q'}\epsilon_{j,q}(\delta'_{j,m,q'})^2 \tag{32}$$

$$P(\epsilon_{j,q}|...) \sim G(ii, jj) \tag{33}$$

where

$$ii = \lambda_0 + M \tag{34}$$

and

$$jj = \upsilon_0 + 0.5 \sum_m (U_{j,m,q} - \gamma_{m,q} - \alpha_{j,m} - \phi_m \delta_{j,m,q})^2 + \quad (35)$$

$$0.5 \sum_m \beta'_{m,q'} (U'_{j,m,q'} - \gamma'_{m,q'} - \alpha_{j,m} - \phi'_m \delta'_{j,m,q'})^2 \quad (36)$$

$$P(\beta'_{m,q'}|...) \sim G(rr', ss') \quad (37)$$

where

$$rr = \kappa_0 + 0.5J \quad (38)$$

and

$$ss = \iota_0 + 0.5 \sum_j \epsilon_{j,q} (U'_{j,m,q'} - \gamma'_{m,q'} - \alpha_{j,m} - \phi'_m \delta'_{j,m,q'})^2 \quad (39)$$

$$P(\alpha_{j,m}|...) \sim N(u, v) \quad (40)$$

where

$$u = \omega_0 \nu_0 + \sum_q \epsilon_{j,q} (U_{j,m,q} - \gamma_{m,q} - \phi_m \delta_{j,m,q}) \quad (41)$$

$$+ \sum_{q'} \beta'_{m,q'} \epsilon_{j,q} (U'_{j,m,q'} - \gamma'_{m,q'} - \phi'_m \delta'_{j,m,q'}) \quad (42)$$

and

$$v = \omega_0 + \sum_q \epsilon_{j,q} + \sum_{q'} \beta'_{m,q'} \epsilon_{j,q} \quad (43)$$

7 Markov Chain Monte Carlo and Diagnostics

Since all priors are conjugates, all posteriors of the Bayesian model can be estimated through a Gibbs sampler [*Casella and George*, 1992], where each unknown is iteratively sampled conditional on the current values of all other unknown parameters.

In each simulation, all unknowns must be initialized. In theory, the MCMC chain should converge to the true target joint distribution regardless of the initial values of the unknowns. However, in order to encourage fast practical convergence, we aim to select well-reasoned values. Table 7 provides the selected starting values for each unknown parameter.

Some values may be relatively far away from the center of their true distributions. Following previous related literature, we initially ran MCMC runs with burnins of size

$N_1 = 250,000$ followed by $N_2 = 250,000$ more samples. We thinned the chain of size $N_2 = 250,000$ by only saving every 50th posterior sample to induce independence between samples, also following the same literature [*Tebaldi et al.*, 2005]. This provided a final posterior of size $N_{final} = 5,000$.

We compared results from this setup to a less computationally expensive one. Specifically, the final default setup for MCMC runs was a burnin of size $N_1 = 500$, a second chain of variable size N_2, and $N_{final} = 10,000$. We achieved $N_{final} = 10,000$ by thinning the N_2 size chain proportional to the effective sample size N_{eff} [*Sturtz et al.*, 2005] of the burnin sample of $\gamma_{m,q'}$, averaged over all m and q'. Specifically, we rounded the ratio $\frac{N_1}{N_{eff}}$ to the nearest integer and took the minimum of this ratio or 10 as a thinning constant TC. Then, to generate $N_{final} = 10,000$, we saved every TC^{th} sample from the post-burnin chain. In practice, we found no notable difference in posterior results between this setup and the one from previous literature [*Tebaldi et al.*, 2005] hence we used this as our default setup unless otherwise noted.

We run standard MCMC diagnostics to check independence of samples and approximate chain convergence. Using the same procedure as above, we again compute the effective sample size of this final thinned posterior sample N_{final}. Those effective sample sizes for the validation scheme runs are shown in Table 8; most are close to 10,000.

We also use the Geweke diagnostic [*Geweke*, 1991] to assess whether the chain has converged to the target posterior distribution. The Geweke diagnostic assumes that the last half of the chain has converged to the target distribution. If the mean of an earlier portion (here, the first $5,000$ values of the final chain) is not significantly different from the mean of the last half (the last 5,000), then it can be reasonable inferred that the chain converged in that portion or earlier [*Geweke*, 1991]. The Geweke diagnostic is computed for each component m, q' of the final posterior distribution of the unknown $\gamma'_{m,q'}$. The diagnostic is a test statistic is a standard Z-score that represents the difference between the two sample means divided by its estimated standard error. The standard error is estimated from the spectral density at zero and so takes into account any residual autocorrelation after thinning (Geweke 1991). Since we do this over all m and q' (in our case a total of $12x25 = 300$ times), we could expect for example 5% of Z-scores to exceed 1.96 in absolute value by chance. Further, since each value of $\gamma'_{m,q'}$ is updated using shared information across M and Q', it could be reasonable to see correlation of Z-scores over

seasons and over ordered return levels. This dependence between tests could distort that 5% percent expectation. For each watershed, we tabulate the percentage of tests where Geweke Z-scores exceed 1.96 in absolute value in Table 8.

8 Statistical assumptions

The design of the Bayesian model involves several statistical assumptions that we define here.

Serial Independence - First, by re-ordering the original block maxima (return levels) in ascending order of intensity, we are effectively making the assumption that there is no serial correlation between temporally ordered return levels. This is a typical assumption in extreme value modeling of block maxima (Coles et al. 1991). The assumption allows us to avoid modeling temporal dependence. To check this assumption, prior to reordering or data processing, we employ the Durbin-Watson serial dependence test [*J. Durbin and Watson*, 1952] in each watershed for return levels of observational data with respect to each season m for the climatology 1950-1974. Figure 6 displays a heatmap of the Durbin-Watson test statistic p-values for each watershed and season m. In most cases, p-values are not significant at a 0.05 level. Only 13 ($\sim$ 6%) p-values are <= 0.05, thus approximately what one would expect to see by random chance.

Stationarity - As within a standard extreme value modeling setting, time-ordered return levels are assumed to be level and trend stationary [*Kwiatkowski et al.*, 1992]. We utilize the Kwiatkowski - Phillips - Schmidt - Shin (KPSS) tests for both types of stationarity in each watershed for return level observations with respect to each season m. Figure 6 also displays a heatmap of the KPSS level and trend stationarity test outcomes. R's base library KPSS test function reports any p-values >= 0.10 simply as being >= 0.10, so these heatmaps only delineate the significant versus insignificant test statistics at 0.05. For the level stationarity test, only 18 ($\sim$ 8%) of cases are significant. Meanwhile only 11 ($\sim$ 5%) of KPSS trend stationarity tests are significant at 0.05. Overall these test results may not be surprising given the research on extreme precipitation trends in the 20th century [*Kunkel*, 2003].

Normality - $U_{j,m,q}$ and $U'_{j,m,q'}$ for any ESM j (or observational dataset indexed by k) are are assumed to be Gaussian conditional on temperature dependence. Recall $U_{j,m,q} = \log(P_{j,m,q} - P_{j,m,q-1})$ for all $q \in [2, ...Q]$. For each observational dataset over

the 1950-1974 climatology ($U_{k,m,q}$) in each watershed, we first utilize the Shapiro Wilk test for normality [*Shapiro and Wilk*, 1965]. More specifically, for each season m, we fit a ordinary least squares linear regression $U_{k,m,q} \sim \delta_{k,m,q}$. We run the Shapiro Wilk test on the residuals from that regression to approximate testing the normality assumption in the Bayesian model. Figure 6 shows a heatmap for the Shapiro Wilk tests. In most cases, p-values are not significant at a 0.05 level. However, the distribution is not uniform; 41 ($\sim$ 19%) p-values are $<=$ 0.05, suggesting there is non-normality in some cases, as we would only expect 5% of p-values to be significance by statistical chance.

We also complement that test with significance tests for skewness and kurtosis computed on those same residuals [*Joanes and Gill*, 1998]. More specifically, we compute a 95% confidence interval from a 1000-iteration ordinary bootstrap for sample skewness and kurtosis statistics. We tabulate the occasions when skewness (kurtosis) is significantly negative (positive) based on this bootstrap procedure. In all cases where there is significance in the skewness tests, the statistics are negative, meaning that the distribution of the residuals of $U_{k,m,q}$ after regression on $\delta_{k,m,q}$ is skewed left. This is owing to the log transformation applied to differentials in ordered block maxima, i.e., $\log(P_{k,m,q} - P_{k,m,q-1})$, which sometimes amplifies outlier behavior of small precipitation values, effectively making the left tail more severe. A total of 33 ($\sim$ 15%) of cases show significant left skew. Only 12 ($\sim$ 6%) of cases exhibit significant negative kurtosis, which falls within the realm of statistical chance. Figure 6 shows heatmaps for significance in the skewness and kurtosis test statistics.

Caution is needed in interpretation in all test results, however, since the tests are not necessarily independent of each other, and behavior of return values could be correlated across watersheds and months.

References

Berelson, W., P. Caffrey, and J. Hamerlinck (2004), Mapping hydrologic units for the national watershed boundary dataset.

Casella, G., and E. George (1992), Explaining the gibbs sampler, *The American Statistician*, *46*(3), 167–174, doi:10.2307/2685208.

Ganguly, A., E. Kodra, S. Chatterjee, A. Banerjee, and H. Najm (2013), Computational data sciences for actionable insights on climate extremes and uncertainty,

in *Computational Intelligent Data Analysis for Sustainable Development*, edited by T. Yu, N. Chawla, and S. Simoff, pp. 127–156, Chapman and Hall/CRC Press, USA.

Geweke, J. (1991), *Evaluating the accuracy of sampling-based approaches to the calculation of posterior moments*, 148, Federal Reserve Bank of Minneapolis.

J. Durbin, J., and G. Watson (1952), Testing for serial correlation in least squares regression, *Biometrika*, *37*(3), 409–428, doi:10.1093/biomet/37.3-4.409.

Joanes, D., and C. Gill (1998), Comparing measures of sample skewness and kurtosis, *Journal of the Royal Statistical Society: Series D (The Statistician)*, *47*(1), 183–189.

Kunkel, K. (2003), North american trends in extreme precipitation, *Natural Hazards*, *29*(2), 291–305, doi:10.1023/A:1023694115864.

Kwiatkowski, D., P. Phillips, P. Schmidt, and Y. Shin (1992), Testing the null hypothesis of stationarity against the alternative of a unit root, *J. of Econometrics*, *54*(1), 159–178, doi:10.1016/0304-4076(92)90104-Y.

Shapiro, S., and M. Wilk (1965), An analysis of variance test for normality (complete samples), *Biometrika*, *52*(3/4), 591–611.

Smith, R., C. Tebaldi, D. Nychka, and L. Mearns (2009), Bayesian modeling of uncertainty in ensembles of climate models, *Journal of the American Statistical Association*, *104*(485), 97–116, doi:10.1198/jasa.2009.0007.

Sturtz, S., U. Ligges, and A. Gelman (2005), R2winbugs: a package for running winbugs from r, *Journal of Statistical Software*, *12*(3), 1–16.

Tebaldi, C., L. Mearns, D. Nychka, and R. Smith (2004), Regional probabilities of precipitation change: A bayesian analysis of multimodel simulations, *Geophys. Res. Lett.*, *31*(24), doi:10.1029/2004GL021276.

Tebaldi, C., R. Smith, D. Nychka, and L. Mearns (2005), Quantifying uncertainty in projections of regional climate change: A bayesian approach to the analysis of multimodel ensembles, *J. Climate*, *18*(10), 1524–1540, doi:10.1175/JCLI3363.1.

Table 1. Modeling Schemes

**Scheme**	**Historical**	**Future**
Validation	1950-1974	1975-1999
End Of Century	1975-1999	2065-2089

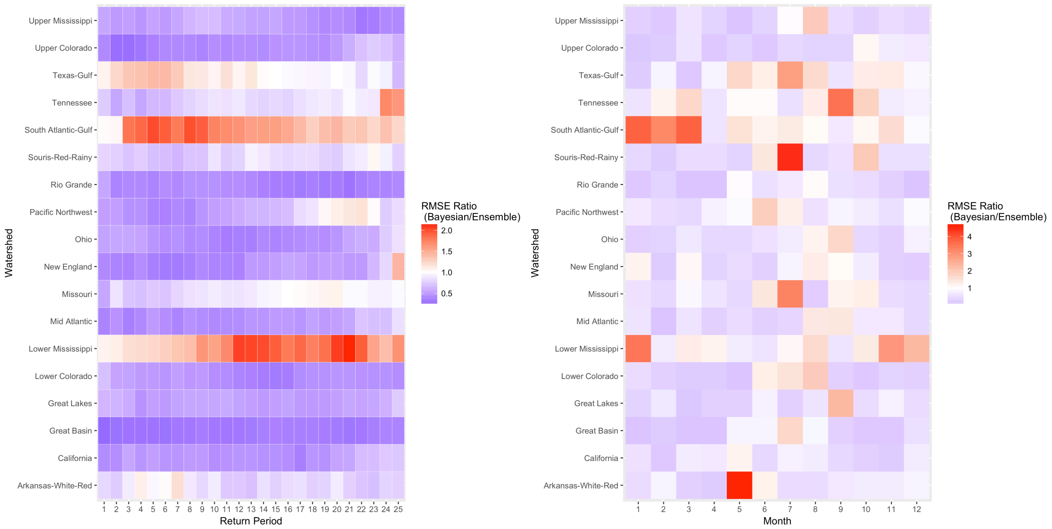

Figure 1. (Left) Validation climatology accuracy of the Bayesian model versus the original ensemble is assessed marginally for each return level $q' \in [1, 2, ...25]$ and each watershed. Values of $\frac{RMSE_{p,q'}}{RMSE_{e,q'}}$ (posterior RMSE over ensemble RMSE, see Appendix) are shown with a heatmap. Blue colored cells are cases where the Bayesian model is more accurate than the original ensemble, and vice versa for red cells. (Right) The same is shown but marginally for every month and watershed. Colors correspond to values of $\frac{RMSE_{p,m}}{RMSE_{e,m}}$.

**ESM Full Name**	**ESM Short Name**	**Resolution (lon x lat)**
CanESM2	canesm2	128 x 64
CCSM4	ccsm4	288 x 192
CESM1-CAM5	cesm1cam5	288 x 192
GFDL-CM3	gfdlcm3	144 x 90
GFDL-ESM2G	gfdlesm2g	144 x 90
inmcm4	inmcm4	180 x 120
IPSL-CM5A-MR	ipslcm5amr	144 x 143
IPSL-CM5B-LR	ipslcm5blr	96 x 96
MIROC5	miroc5	256 x 128
MIROC-ESM	mirocesm	128 x 64
MIROC-ESM-CHEM	mirocesmchem	128 x 64
MPI-ESM-LR	mpiesmlr	192 x 96
MPI-ESM-MR	mpiesmmr	192 x 96
MRI-CGCM3	mricgcm3	320 x 160
NorESM1-M	noresm1m	144 x 96

Table 2. CMIP5 Earth System Models (ESMs) included in this study. For each ESM, we use only model runs labeled r1i1p1. For all future climatologies, we use ESM outputs conditioned on greenhouse gas trajectory scenario RCP8.5.

Table 3. USGS HU2 Watersheds

**ID**	**Watershed**
1	Lower Mississippi
2	Tennessee
3	Pacific Northwest
4	Missouri
5	Arkansas-White-Red
6	Souris-Red-Rainy
7	Mid Atlantic
8	Upper Colorado
9	Lower Colorado
10	Ohio
11	Upper Mississippi
12	New England
13	Great Basin
14	South Atlantic-Gulf
15	Texas-Gulf
16	Rio Grande
17	California
18	Great Lakes

**Parameter**	**Interpretation**
**Data Terms**	
$Z_{k(j),m}$	Smallest (N=1) year return value
$U_{k(j),m,q(q')}$	Logged differential between successively ranked return period values (e.g., N=2 minus N=1)
$\delta_{k(j),m,q}$	Difference between same day temperature of successively ranked return period values
**Fixed Terms**	
σ_k	Weight (inverse variance) for $q = 1$ observations
$\epsilon_{k,q}$	Weight (inverse variance) for $q = q' > 1$ observations, by return period $q = q'$
τ_k	Observational weight scaling parameter (modulates the weighting of observations relative to ESMs)
**Unknown Parameters**	
C_m	True $q = 1$ precipitation (historical climatology)
C'_m	True $q' = 1$ precipitation (future climatology)
$CBIAS_j$	Bias term for true $q = 1$ precipitation for ESM j
$\sigma_k(j)$	Weight (inverse variance) for $q = 1$ observations or ESMs
θ	Variance scaling parameter for future $q' = 1$ precipitation
$\gamma_{m,q}$	True historical logged differences between successive ranked precipitation (e.g., N=2 minus N=1)
$\gamma'_{m,q'}$	True future logged differences between successive ranked precipitation (e.g., N=2 minus N=1)
ϕ_m	Historical temperature dependence parameter
ϕ'_m	Future temperature dependence parameter
$\alpha_{j,m}$	Bias term for true $q > 1$ precipitation for ESM j
$\epsilon_{j,q}$	Weight (inverse variance) for $q = q' > 1$ ESMs, by return period $q = q'$
$\beta'_{m,q'}$	Variance scaling parameter for future $q' > 1$ precipitation

Table 4. Data terms, fixed terms, and Bayesian unknown parameters along with brief interpretations.

**Parameter**	**Value**	**Notes**
$C_{0,m}$	0	
$\sigma_{0,m}$	0.01	Relatively small weight makes $C_{0,m}$ less informative
δ_0	0	Presume ESMs are unbiased
ρ_0	1	
α_0	0.1	Uninformative prior for ESM weights
β_0	0.1	
ζ_0	1	**
η_0	10	**
γ_0	1e-7	Presume a small increase in subsequent block maxima
ϵ_0	1	
ν_0	0	Presume ESMs are unbiased
ω_0	1	Relatively large weight to influence bias closer to 0
ϕ_0	0	Presume no linear relationship with temperature
ξ_0	1	Relatively small weight makes ϕ_0 less informative
λ_0	0.1	Uninformative prior for ESM weights
υ_0	0.1	
κ_0	10	**
ι_0	25	**

Table 5. Prior Parameters and Values. Selected priors generally work well in practice. Those rows with ** indicates that we observed relative sensitivity of results to choice of their values. For these, we explore a range of values in a prior sensitivity study in the two regions where we do extensive analysis. Priors without ** exert relatively little influence as long as the choices for their values are reasonable.

Table 6. Selected priors and candidate values.

Parameter	Candidate Values
ζ_0	1, 10, 25, 50, 75
η_0	1, 10, 25, 50, 75
κ_0	1, 10, 25, 50, 75
ι_0	1, 10, 25, 50, 75

Table 7. Parameter Starting Values

Unknown	Value	Notes
C'_m	2	Starting point of exp(2) $=\sim$ $7.4\frac{mm}{day}$ for smallest block maxima ($q = q' = 1$)
$CBIAS$	0	Presume no ESM bias
σ_j	1	
θ_j	1	Presume consensus could be equally as important as skill
$\gamma_{m,q}, \gamma'_{m,q'}$	0.05	
ϕ_m, ϕ'_m	0	Presume no temperature-precipitation dependence
$\alpha_{j,m}$	0	Presume no ESM bias
$\epsilon_{j,q}$	1	
$\beta'_{j,q'}$	1	Presume consensus could be equally as important as skill

**ID**	**Watershed**	**Effective** N_{final}	% **Geweke** $\|Z\| \leq 1.96$
1	Lower Mississippi	10000	100
2	Tennessee	10000	100
3	Pacific Northwest	9311	99.7
4	Missouri	7071	92.7
5	Arkansas-White-Red	9460	100
6	Souris-Red-Rainy	8354	92
7	Mid Atlantic	9448	95.3
8	Upper Colorado	7812	100
9	Lower Colorado	8917	100
10	Ohio	9583	100
11	Upper Mississippi	9009	100
12	New England	9512	95.7
13	Great Basin	8221	96.3
14	South Atlantic-Gulf	9561	100
15	Texas-Gulf	8457	100
16	Rio Grande	8996	100
17	California	8833	95.3
18	Great Lakes	9523	100

Table 8. Watershed level MCMC diagnostics are displayed. The effective final sample size of after thinning is computed as Effective N_{final}. The percentage of Geweke test Z-scores per watershed that are <= |1.96| is displayed in the column labeled Non-Sig. Geweke Z-Scores. Here effective sample size refers to the effective sample size of the MCMC output. Because the sequence of samples that come from the MCMC are autocorrelated, effective sample size estimates the true effective number of samples after thinning (only saving every X samples). This is a useful diagnostic in classic MCMC modeling. If effective sample size is too low, statistics from the posterior distribution could be biased.

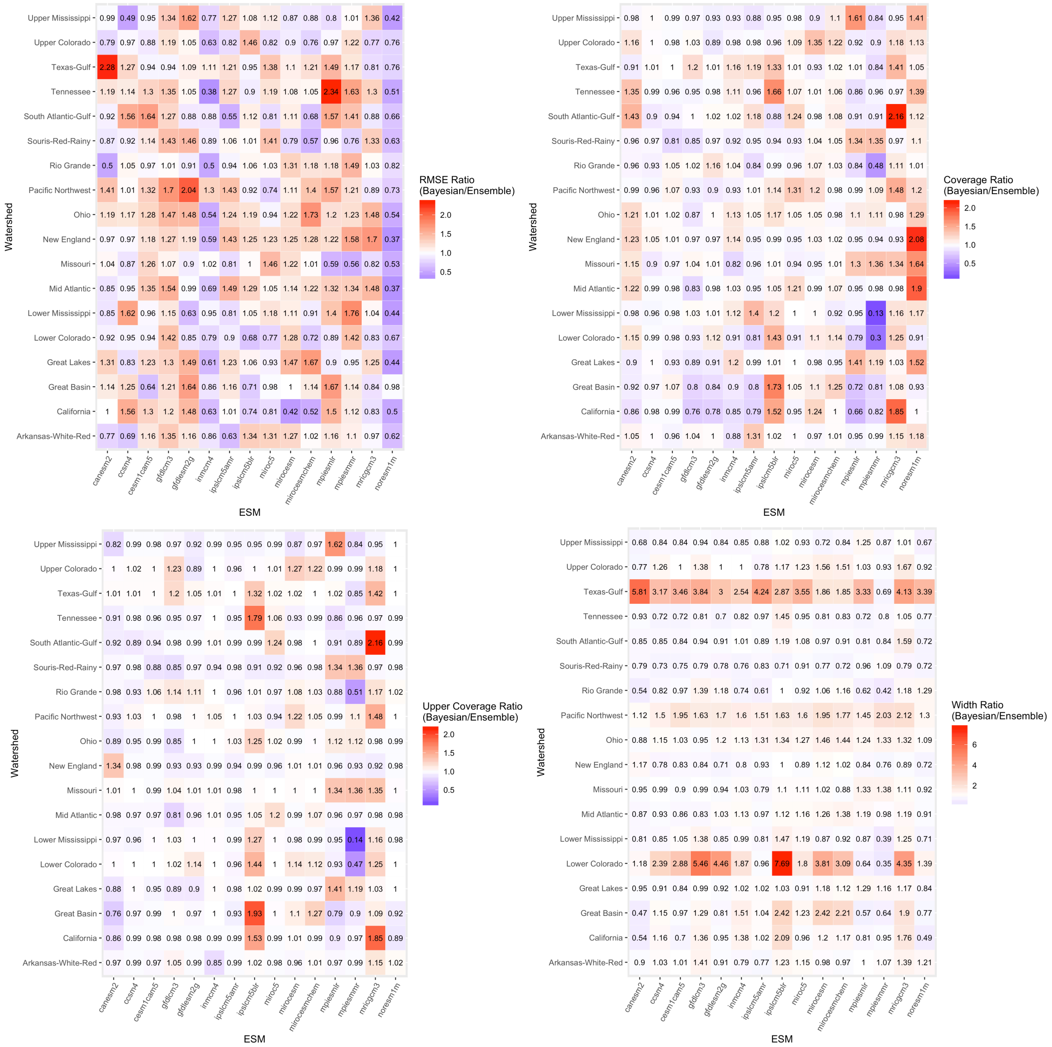

Figure 2. In each watershed, a cross validation exercise is conducted wherein each ESM is iteratively left out and treated as a proxy for observed data, where the historical and future climatologies are 1975-1999 and 2065-2089, respectively. (Top left) Ratios $\frac{RMSE_p}{RMSE_e}$ are shown via heatmap for each held-out ESM and over each watershed, where RMSE is averaged over return periods and seasons. (Top right) Coverage ratios $\frac{cov_p}{cov_e}$ are displayed. (Bottom left) Upper coverage ratios $\frac{cov_p^u}{cov_e^u}$ are displayed. (Bottom left) Width ratios $\frac{W_p}{W_e}$ are depicted.

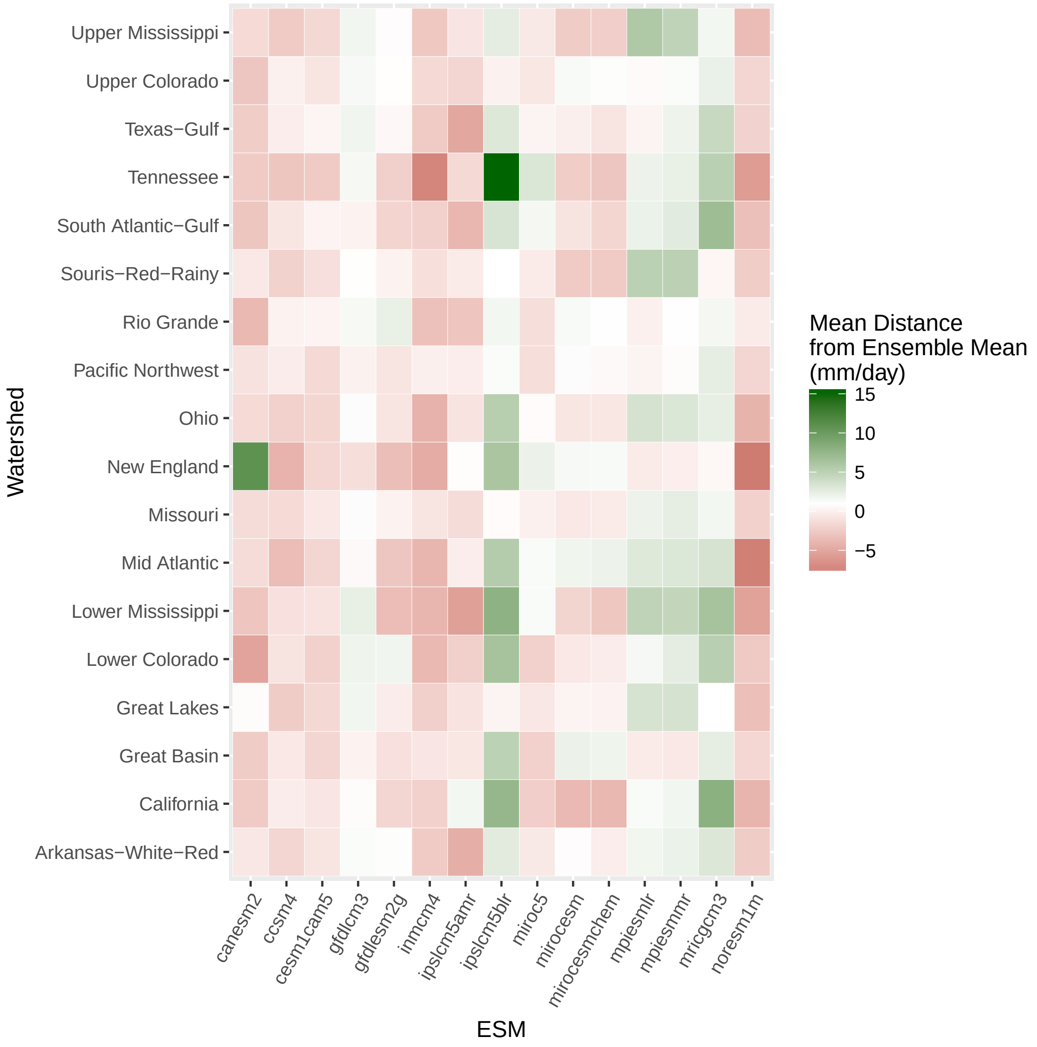

Figure 3. Average distance from the ensemble mean is calculated for each ESM over each watershed for 2065-2089. Specifically, all precipitation depths are subtracted from the mean precipitation over all seasons and return periods for a given overall ESM and watershed average. Those differences are then averaged at per watershed and ESM. In the heatmap, brown cells are those where an ESM on average is “drier” and green wetter than the average of the ensemble. These differences are moderately and positively correlated to the RMSE, upper coverage, and width ratios (but not the coverage ratio) displayed in Figure 2.

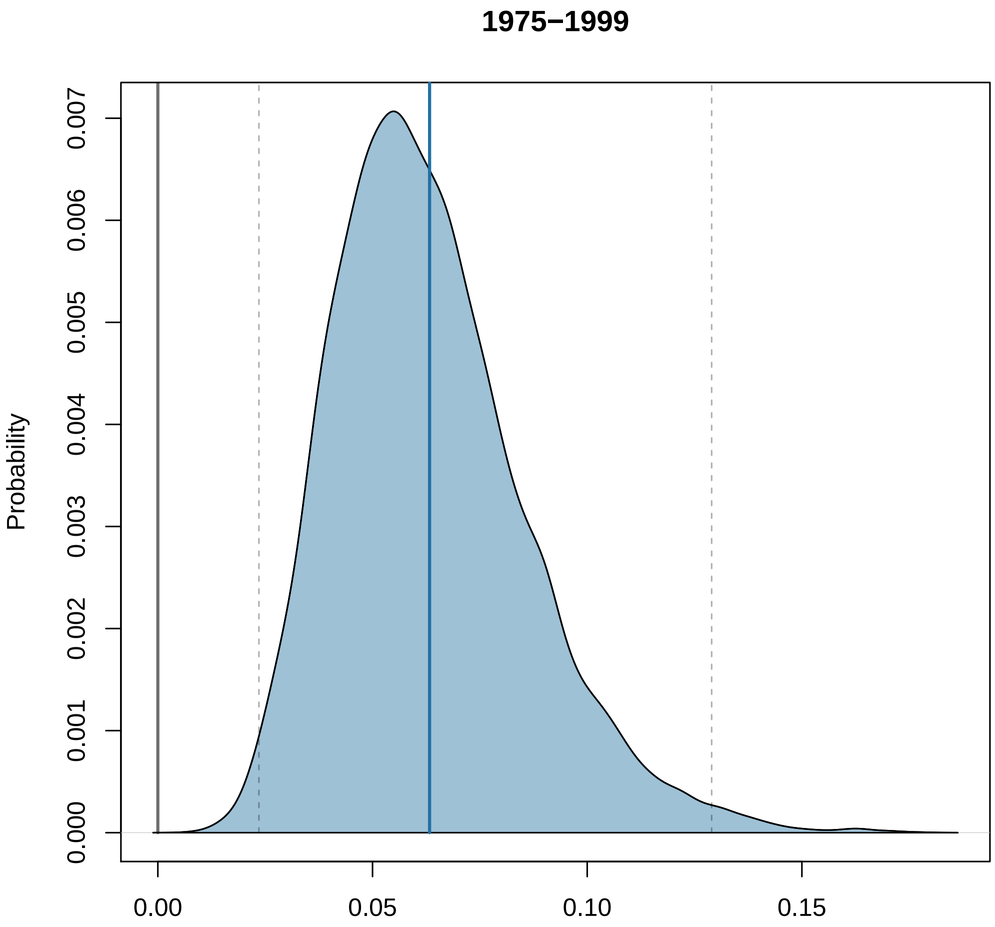

Figure 4. Posterior of θ, a future variance scaling parameter for $q, q' = 1$, is shown for the validation scheme model run (1950-1974 as training, 1975-1999 as validation) in the Ohio watershed. Values are substantially less than 1, meaning that consensus is favored less than skill in weighting ESMs for determining the posteriors of C'_m.

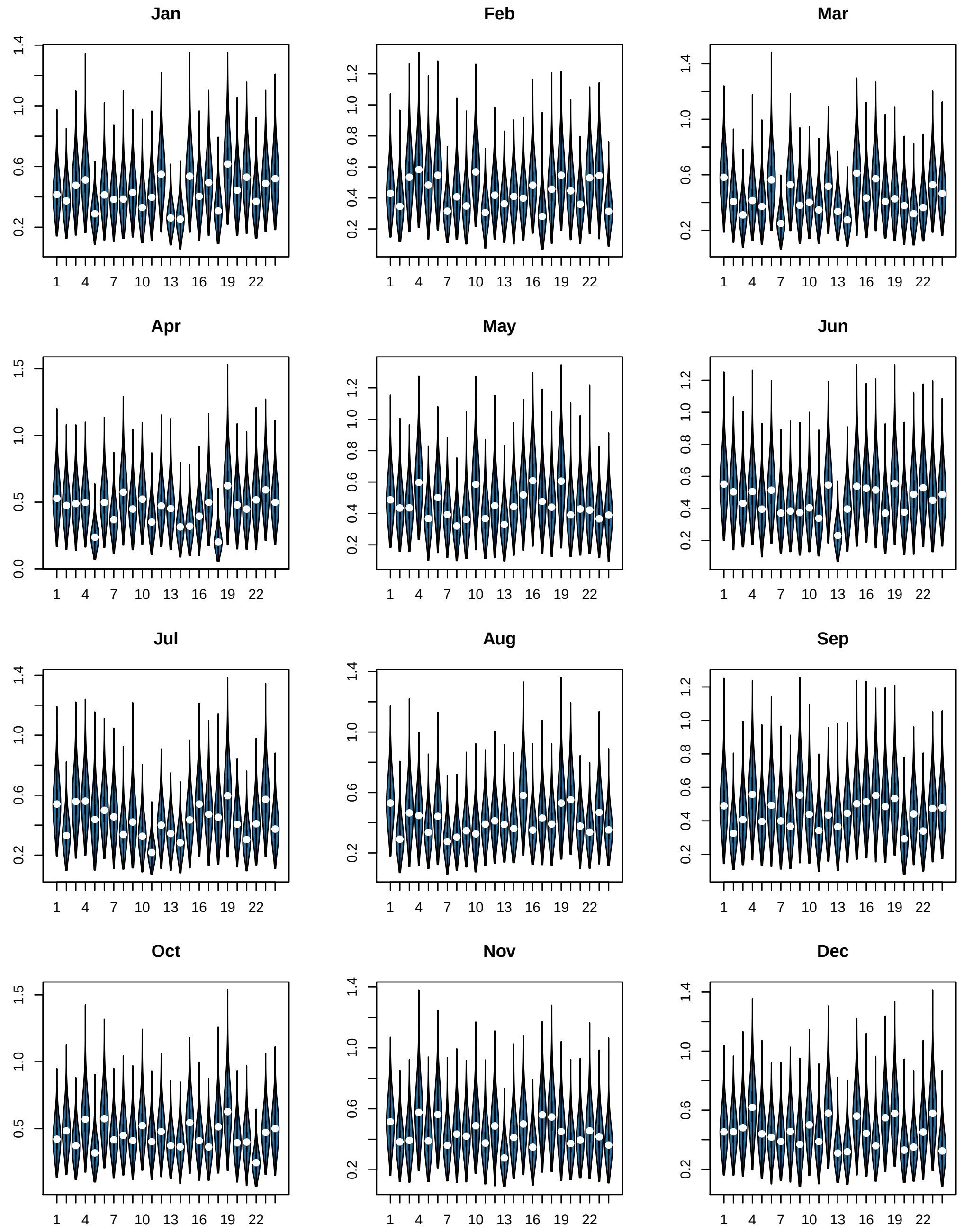

Figure 5. Posteriors of $\beta'_{m,q'}$ are shown via violin plots for the validation scheme model run in the Ohio watershed. Horizontal axes range from 1 to 24, which map to $q, q' \in [2, ...Q = Q' = 25]$.

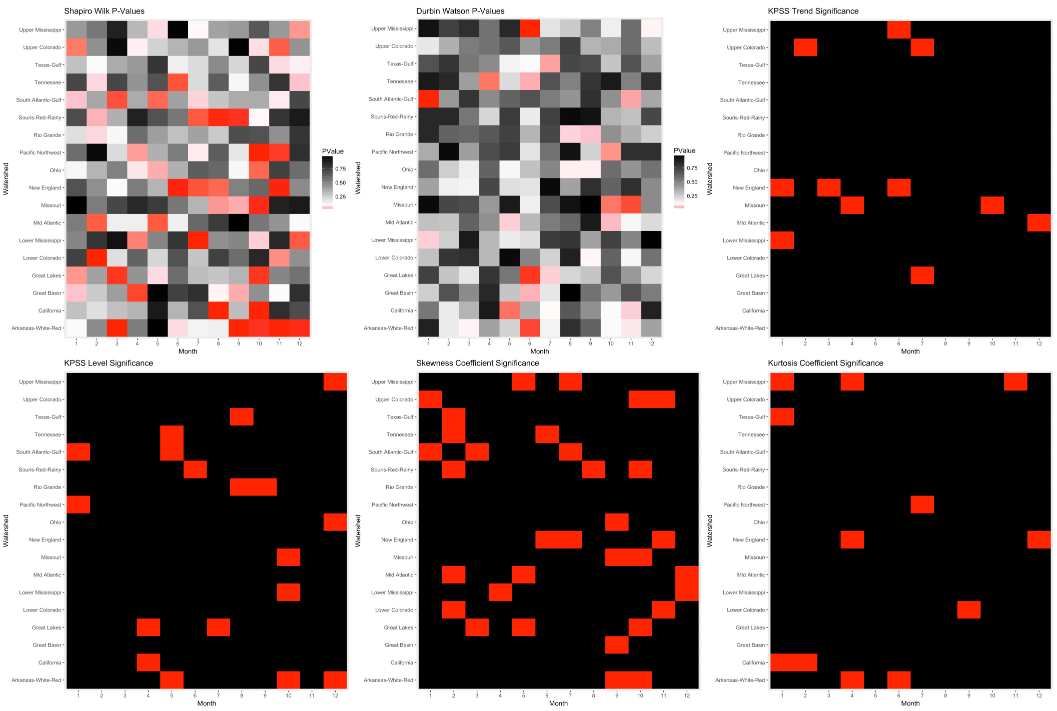

Figure 6. (Top left) Shapiro Wilk normality tests are applied to observational data return levels across each watershed with respect to each season for the climatology 1950-1974. The heatmap displays p-values from those tests. Red coloring indicates significance at 0.05, pink at 0.10, and grayscale is used for p-values above 0.10.(Top middle) The Durbin-Watson serial dependence test is applied (before re-ordering based on rank) in each watershed for observational data return levels with respect to each season m for the climatology 1950-1974. A heatmap of the Durbin-Watson test statistic p-values is displayed by watershed and month. The color scale follows the same pattern as for the Shapiro Wilk tests. (Top right) Prior to reordering, KPSS tests are employed to test for level stationarity across all watersheds and months to observational return levels from 1950-1974. In this heatmap, red coloring indicates significant trend non-stationarity at a 0.05 level, and black insignificant. (Bottom left) The same as in the top right but for level stationarity. (Bottom middle) For each month and watershed, observational return levels from 1950-1974 are tested for significant skewness. Specifically, a standard 10,000 member bootstrapped distribution of the sample skewness coefficient is computed from $U_{k,m,q}$. From this a 95% confidence interval is estimated. In every instance where that confidence interval does not include 0, a non-black square is entered in the heatmap. All red squares indicate significant negative skew. There are no cases where significant positive skew is found. (Bottom right) The same is shown as in the bottom left but for kurtosis. The same bootstrapped values are used for testing significance of both skewness and kurtosis.

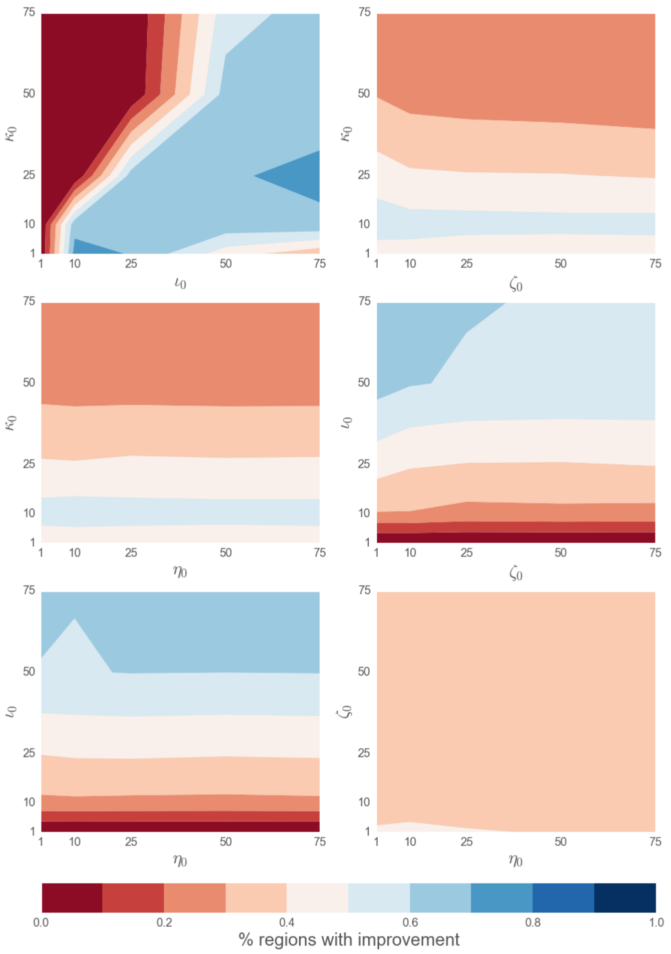

Figure 7. Prior sensitivity is examined across all 18 HU2 watersheds for the parameters κ_0, ι_0, η_0, and ζ_0. Percent of regions where $RMSE_p \leq RMSE_e$ is depicted with the contour plots for all pairwise combinations of those four parameters. Choice of κ_0 and ι_0 exert the largest influence over model performance; this can be seen most clearly in the upper left plot.

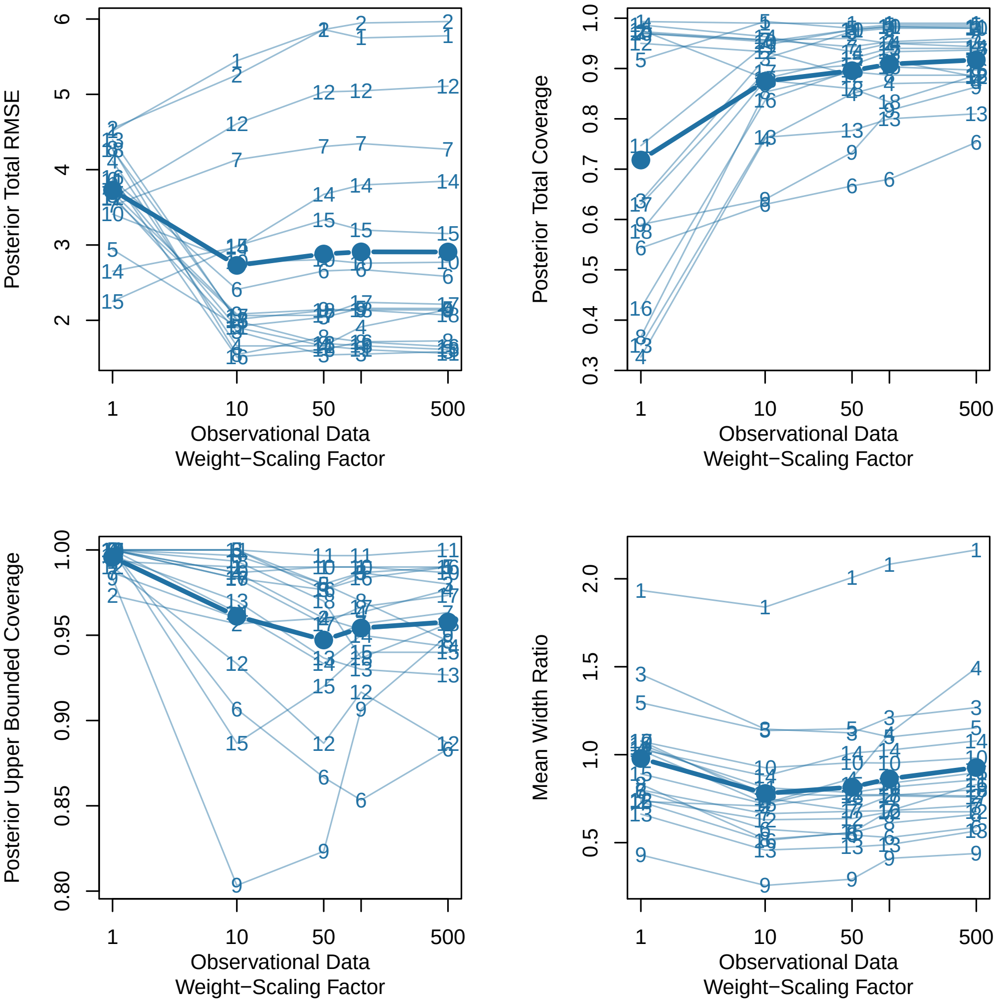

Figure 8. Posterior total RMSE ($RMSE_p$), posterior coverage (cov_p), upper posterior coverage (cov_p^u), and the ratio of average posterior distribution width over average ensemble width ($\frac{W_p}{W_e}$) are plotted as a function of τ_k, which is set at values of 1, 10, 50, 100, and 500. For each subplot, opaque numbered blue lines represent individual watersheds, and the thicker blue line is the mean of those lines. Numbers map to watersheds from Table 3. The quantities cov_p, cov_p^u, and $\frac{W_p}{W_e}$ are computed via a 99% credible interval. $RMSE_p$ decreases until $\tau_k = 100$. cov_p increases but appears asymptote at $\tau_k = 100$. cov_p^u is generally insensitive to τ_k but does decrease slightly with larger values of τ_k. The width ratio $\frac{W_p}{W_e}$ is apparently relatively insensitive to τ_k.

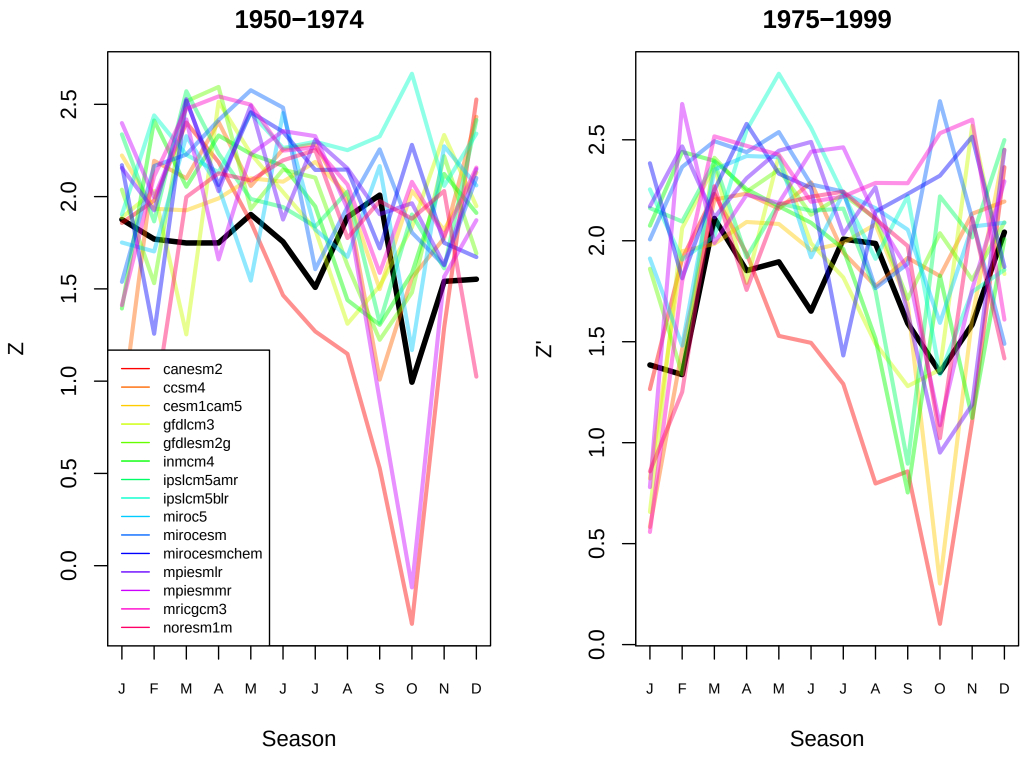

Figure 9. (Log-transformed $q = 1$ return levels are shown for observations ($Z_{k,m,q=1}$ and $Z'_{k,m,q'=1}$) and ESMs ($Z_{j,m,q=1}$ and $Z'_{j,m,q'=1}$) for the 1950-1974 and 1975-1999 climate regimes. The black line shows the observations and the colored lines the ESMs.

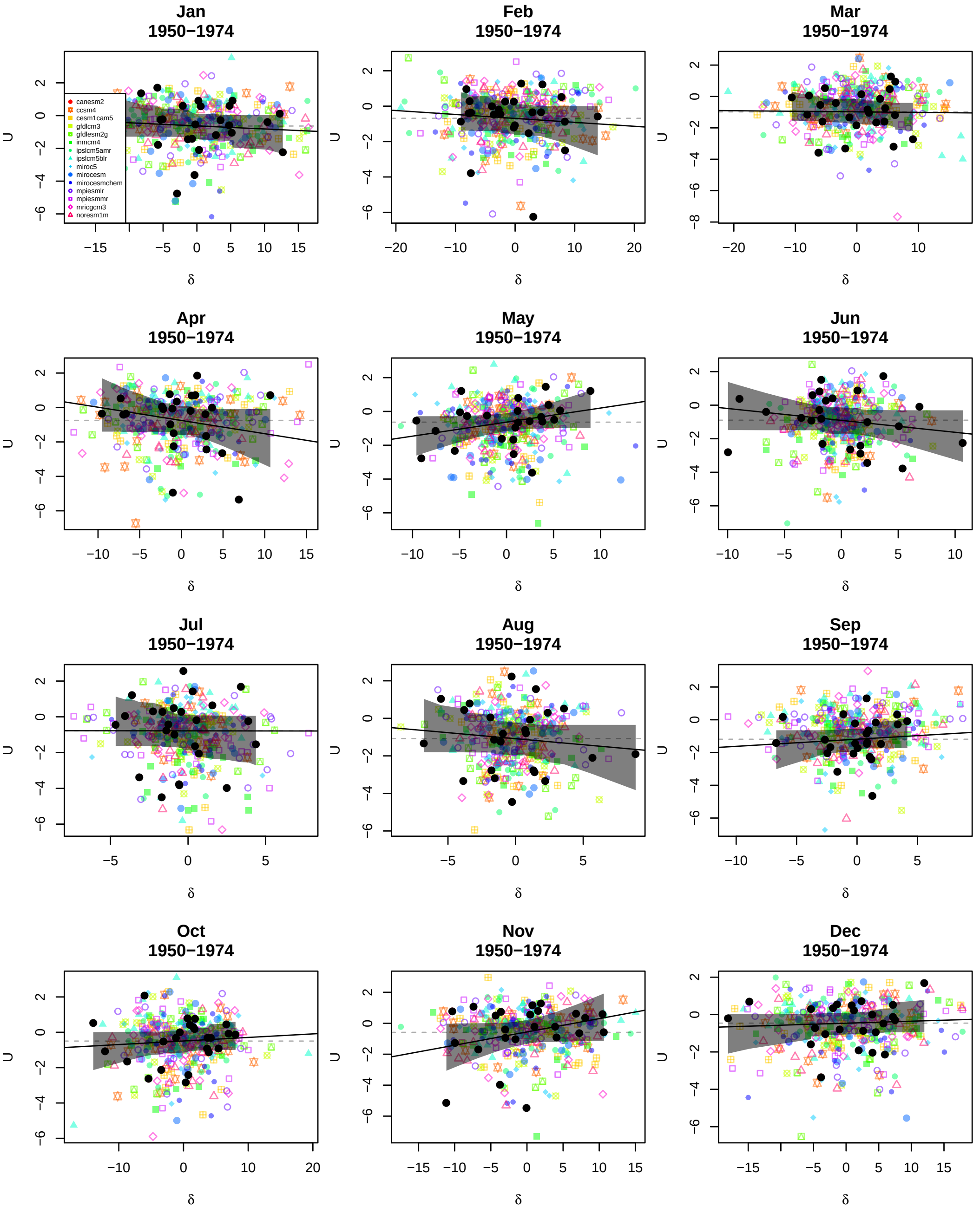

Figure 10. In the Ohio watershed, for each month, $U_{j,m,q}$ are plotted against corresponding values of $\delta_{j,m,q}$. Black points are observations. Black lines and bounds are least squares lines and 95% prediction interval bounds, representing the observed temperature-precipitation. Each color and point type combination represents data for one ESM.

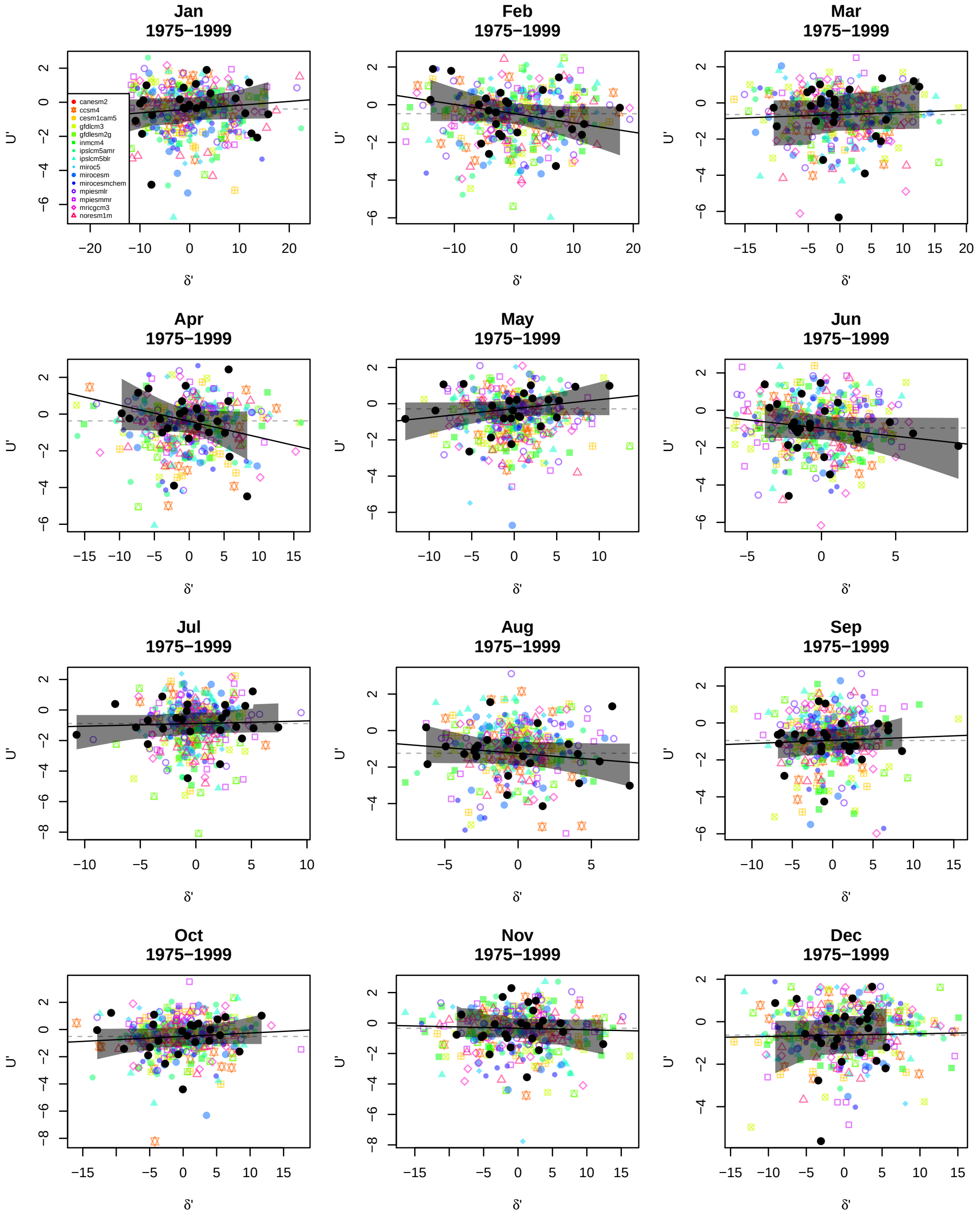

Figure 11. The same as Figure 10 is shown but for 1975-1999, i.e., $U'_{j,m,q'}$ and $\delta_{j,m,q'}$.

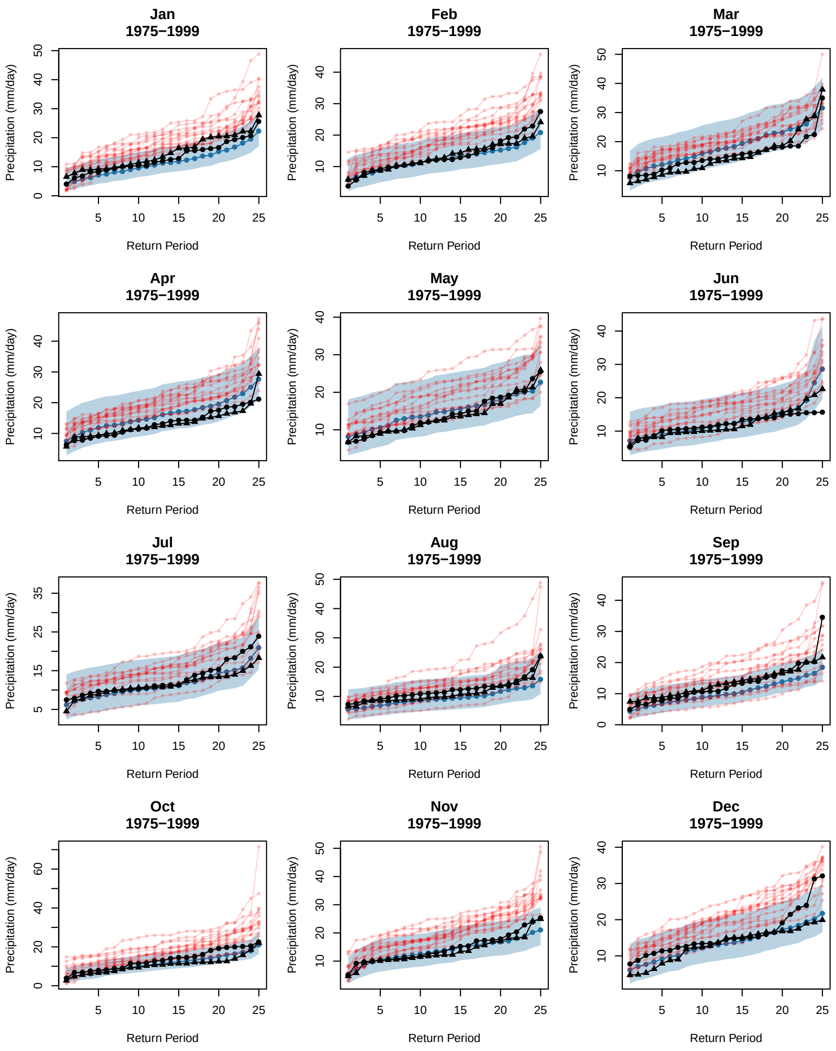

Figure 12. Validation regime posterior distributions for $P'_{p,m,q',n\in[1,2,...N_{final}]}$ are shown for each month in the Ohio watershed. Black dots are return levels of held out 1975-1999 observations, and black triangles are the same but from 1950-1974. Larger blue dots represent the posterior mean for each order statistic q' (i.e., return period). Blue opaque bounds represent a 99% credible interval for each q' and m. Red points show original ESM return values.

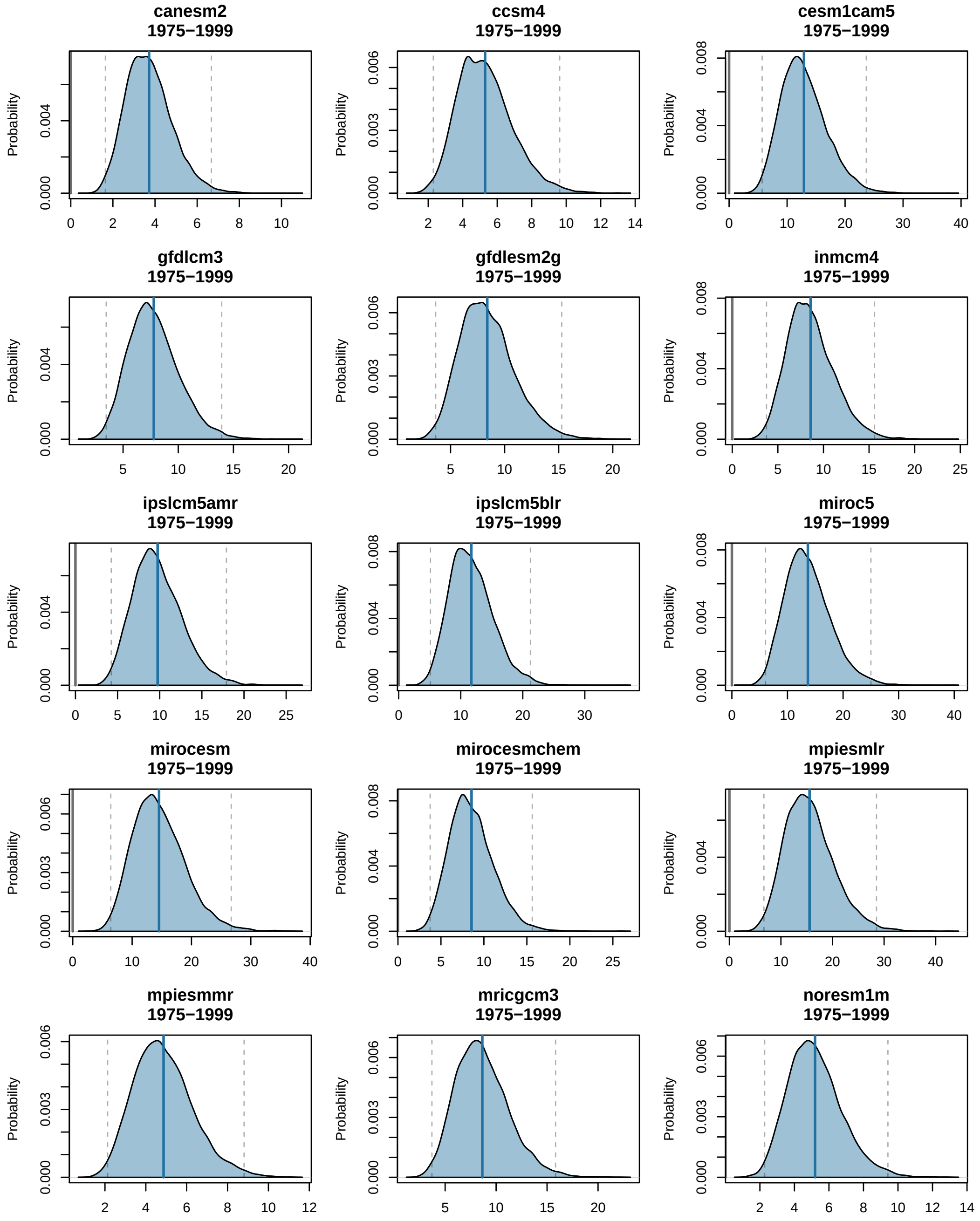

Figure 13. Posteriors of σ_j are shown for the validation scheme model run in the Ohio watershed. Vertical blue lines indicate posterior means and dashed vertical gray lines show 99% credible interval bounds.

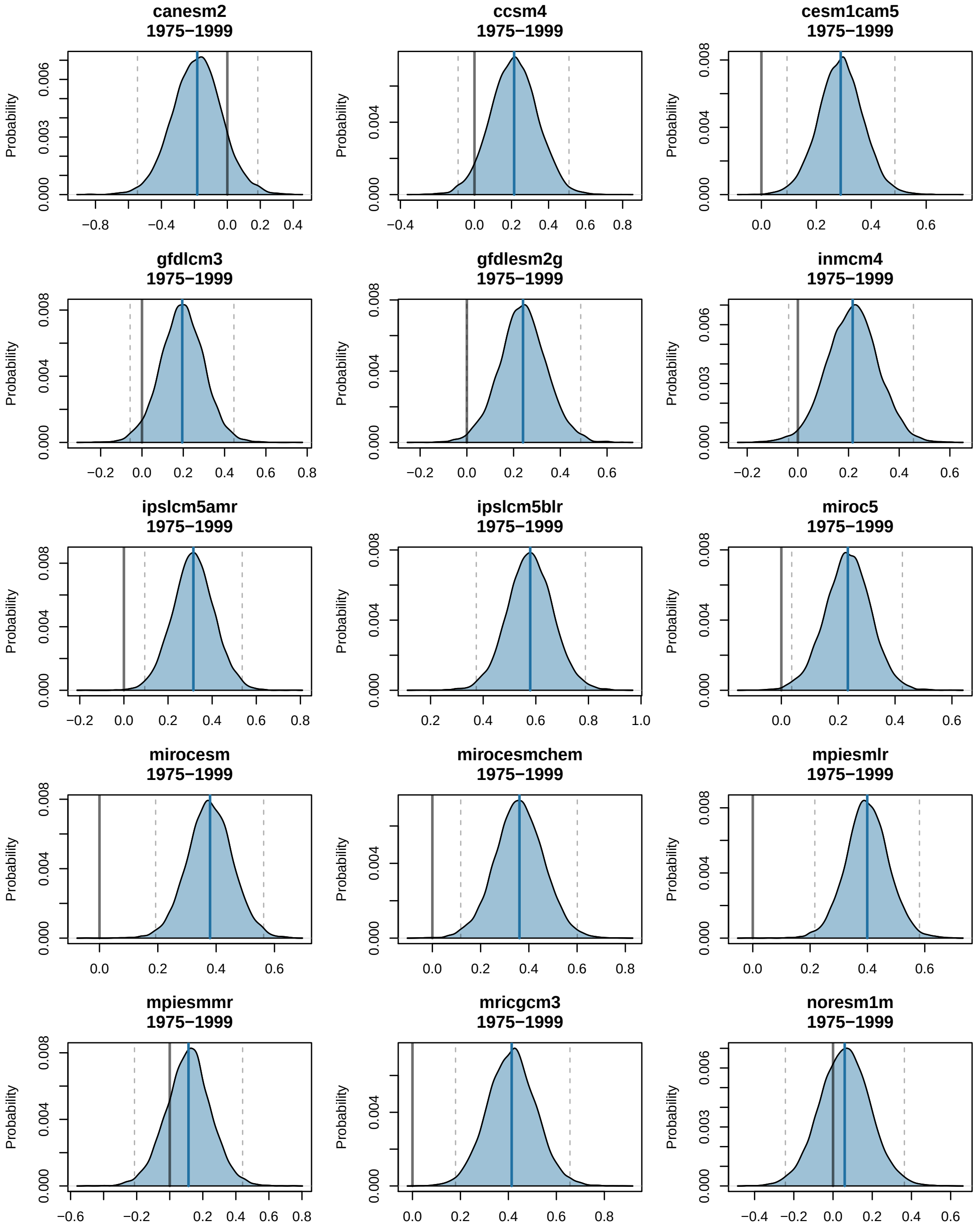

Figure 14. Posteriors of $CBIAS_j$ are shown for the validation scheme model run in the Ohio watershed. Vertical blue lines indicate posterior means and dashed vertical gray lines show 99% credible interval bounds.

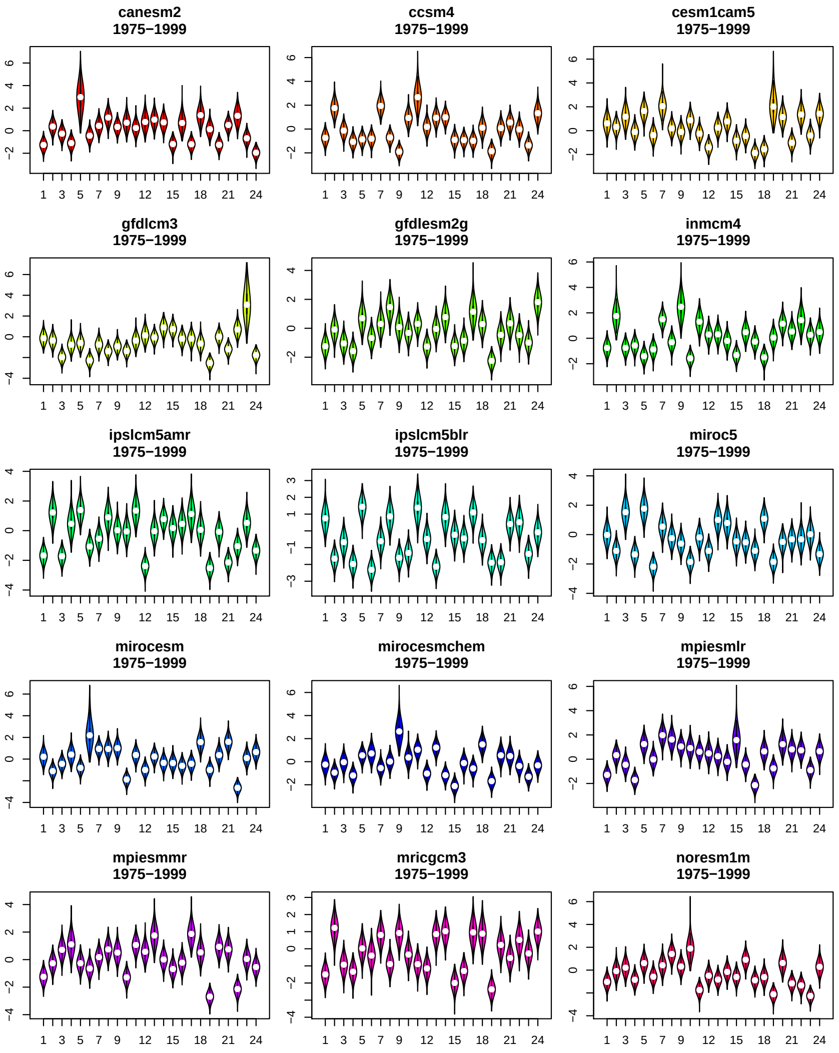

Figure 15. Posteriors of $\epsilon_{j,q}$ (log scale) are depicted with violin plots for the validation scheme model run in the Ohio watershed. Horizontal axes range from 1 to 24, which map to $q, q' \in [2, ...Q = Q' = 25]$. The log scale is used to temper the visual effect of occasional large values. Values of $\epsilon_{j,q}$ can occasionally be large but typically smaller than $\epsilon_{k,q}$.

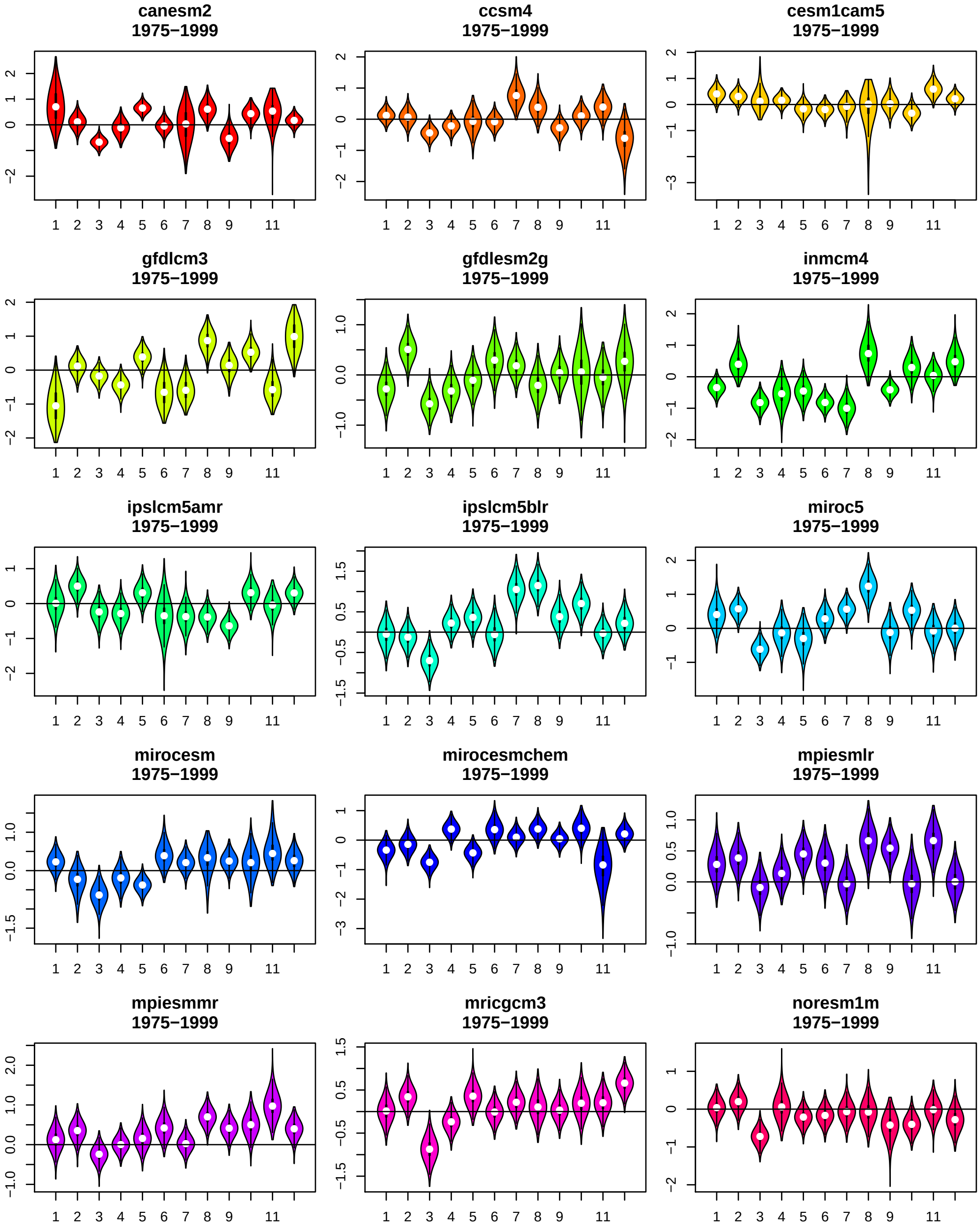

Figure 16. Posteriors of $\alpha_{j,m}$ are shown via violin plots for the validation scheme model run in the Ohio watershed. Horizontal axes range from 1 to 12, reflecting all 12 months.

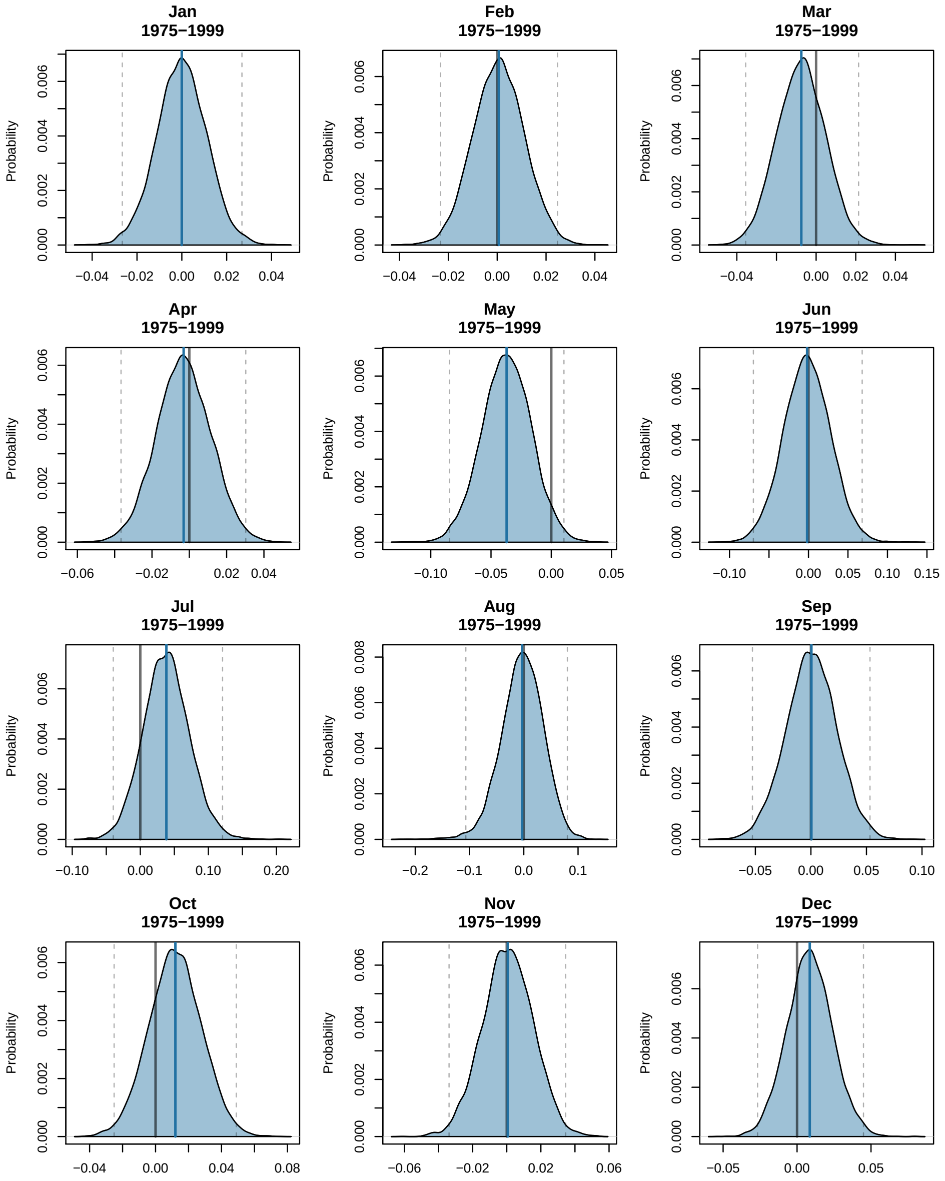

Figure 17. Posteriors of ϕ'_m shown for each month for the validation scheme model run in the Ohio watershed. The vertical blue lines represent the posterior mean, vertical solid gray lines are all at 0, and vertical dashed lines represent 99% credible interval bounds.
